# Genetics of psycho-emotional well-being: genome-wide association study and polygenic risk score analysis

**DOI:** 10.3389/fpsyt.2023.1188427

**Published:** 2024-01-24

**Authors:** Anna Yurievna Yakovchik, Darya V. Tolynyova, Daria A. Kashtanova, Ekaterina R. Sutulova, Mikhail V. Ivanov, Alexandra A. Mamchur, Veronika V. Erema, Lorena R. Matkava, Mikhail V. Terekhov, Antonina M. Rumyantseva, Olga I. Blinova, Aleksandra I. Akinshina, Sergey I. Mitrofanov, Vladimir S. Yudin, Valentin V. Makarov, Anton А. Keskinov, Sergey A. Kraevoy, Sergey M. Yudin

**Affiliations:** Federal State Budgetary Institution Centre for Strategic Planning and Management of Biomedical Health Risks of the Federal Medical Biological Agency, Moscow, Russia

**Keywords:** anxiety, anxiety symptoms, genome-wide association study, HADS, polygenic risk score

## Abstract

**Background:**

Psycho-emotional well-being is essential for living a life of satisfaction and fulfillment. However, depression and anxiety have become the leading mental health issues worldwide, according to the World Health Organization. Both disorders have been linked to stress and other psychological factors. Their genetic basis remains understudied.

**Methods:**

In 2020–2021, the psycho-emotional well-being of 30,063 Russians with no known psychiatric history was assessed using the Hospital Anxiety and Depression Scale (HADS) for general mental health and the HADS subscale A (anxiety) for anxiety. Following the original instructions, an anxiety score of ≥11 points was used as the anxiety threshold. A genome-wide association study was performed to find associations between anxiety and HADS/HADS-A scores using linear and logistic regressions based on HADS/HADS-A scores as binary and continuous variables, respectively. In addition, the links between anxiety, sociodemographic factors (such as age, sex, and employment), lifestyle (such as physical activity, sleep duration, and smoking), and markers of caffeine and alcohol metabolism were analyzed. To assess the risk of anxiety, polygenic risk score modeling was carried out using open-access software and principal component analysis (PCA) to simplify the calculations (ROC AUC = 89.4 ± 2.2% on the test set).

**Results:**

There was a strong positive association between HADS/HADS-A scores and sociodemographic factors and lifestyle. New single-nucleotide polymorphisms (SNPs) with genome-wide significance were discovered, which had not been associated with anxiety or other stress-related conditions but were located in genes previously associated with bipolar disorder, schizophrenia, or emotional instability. The *CACNA1C* variant *rs1205787230* was associated with clinical anxiety (a HADS-A score of ≥11 points). There was an association between anxiety levels (HADS-A scores) and genes involved in the activity of excitatory neurotransmitters: *PTPRN2 (rs3857647)*, *DLGAP4 (rs8114927)*, and *STK24 (rs9517326)*.

**Conclusion:**

Our results suggest that calcium channels and monoamine neurotransmitters, as well as SNPs in genes directly or indirectly affecting neurogenesis and synaptic functions, may be involved in the development of increased anxiety. The role of some non-genetic factors and the clinical significance of physiological markers such as lifestyle were also demonstrated.

## 1 Introduction

Psycho-emotional well-being is essential for functioning effectively in all areas of life. Anxiety and depression are the most common mental disorders worldwide. According to the World Health Organization (WHO), approximately 264 million people suffered from anxiety disorders in 2017. In 2015, the overall prevalence of mental illness was on the rise, and the number of people with anxiety disorders increased by 14.9% since 2005 (1). The COVID-19 pandemic sparked a global mental health crisis in 2020, with 25% of the world’s population affected by a phobia or an anxiety disorder (2).

Anxiety is of particular interest among all stress-related mental disorders due to its complex psychophysiological nature. Fear and anxiety are natural physiological responses to certain situations. However, anxiety that is overwhelming, persistent, or disproportionate to the actual threat is a psychological condition that can disrupt personal, social, and professional lives.

Anxiety is a term used in psychiatry to describe a variety of disorders. The difference between them is not obvious. Furthermore, anxiety is often associated with other mental disorders, such as depression (3). Some people are anxiety sensitive; they are afraid of feeling anxious and the physical sensations that come with it. Anxiety sensitivity is generally considered to be a personality trait.

According to the National Institute of Mental Health (NIMH), 31.1% of US adults will experience at least one episode of anxiety in their lifetime (4). Despite its prevalence, there are still gaps in our understanding of the causes and underlying mechanisms of anxiety. Its development appears to be triggered by biopsychosocial factors. When stressed or traumatized, genetically predisposed individuals may exhibit clinically significant anxiety symptoms similar to those of other mental disorders (5).

Further research is needed to understand the role of genetic factors in the development of anxiety and the predisposition to excessive pathological anxiety and anxiety sensitivity. According to genetic epidemiological studies, 30–50% of all anxiety disorders are hereditary (6). However, many authors believe that all mental illnesses, not just anxiety, have a genetic component.

One of the least understood aspects of anxiety is the genetic mechanisms underlying its development and progression. Attempts have been made to examine candidate genes encoding monoamine transporters (7, 8). Several cohort studies, such as a recent GWAS involving over 25,000 people with anxiety disorders or a history of episodic anxiety, have examined the genetic basis of anxiety. Five loci of genome-wide significance have been found, including an intergenic region (IGR) on chromosome 9, previously associated with neuroticism, and a locus overlapping neurotrophic receptor tyrosine kinase type 2 (*NTRK2*), the receptor gene for brain-derived neurotrophic factor (BDNF) (9). Anxiety disorders have been found to have a significant genetic component. However, additional research is needed for a more comprehensive understanding of the relationship between an individual’s genetics and the development and clinical manifestations of anxiety.

Most research on the genetics of anxiety has focused on specific disorders. Therefore, people who are prone to anxiety but do not have a clinically diagnosable pathological condition are of particular interest. Levey et al. used the Generalized Anxiety Disorder 2-item (GAD-2) screening scale to assess anxiety in 200,000 participants in the Million Veterans Program (MVP, USA) (10). They performed a GWAS based on GAD-2 scores and discovered five significant signals for European Americans and one for African Americans throughout the genome. The strongest signals were found on chromosome 1, in the intron of *MAD1L*, and near *SATB1* and *SATB1-ASI*. Approximately 90% of patients were male; however, one of the strongest signals was in the intron of the estrogen receptor gene (*ESR1*), previously implicated in anxiety-like behavior in animal models. These results suggest that the mere presence of symptoms could be of value for identifying the genetic factors underlying anxiety. In addition, most anxiety patients are diagnosed with a mild disorder (43.5%). Many people with anxiety are unlikely to seek medical attention and stay outside the scope of anxiety studies.

**Figure 1:**
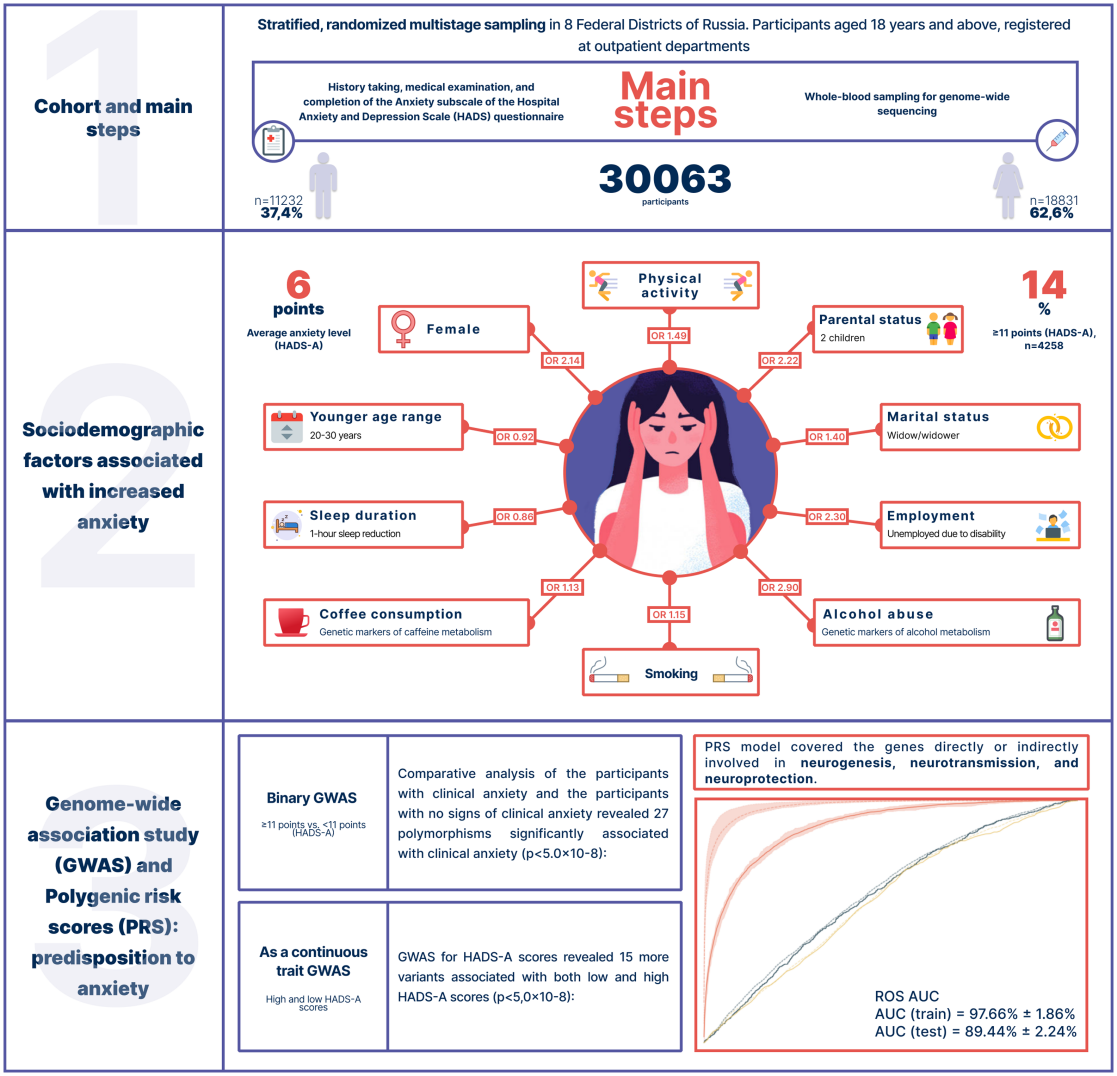
Study design, cohort characteristics, and key results.

Unfortunately, there is not much research on the psycho-emotional well-being of the Russian population. Therefore, assessing the prevalence of anxiety in Russia is a challenging task. To better understand the genetics of anxiety, we evaluated data from an epidemiological study (conducted between 2020 and 2021) of 30,063 Russian adults who had never sought psychiatric treatment. To assess overall mental distress and anxiety, we used the Hospital Anxiety and Depression Scale (HADS) and its HADS-A subscale, a widely used and internationally validated screening tool for anxiety and depression. We examined the participants’ caffeine and alcohol consumption, sleep duration, and physical activity as potential factors contributing to increased anxiety levels. We also analyzed the effects of genetic variants associated with alcohol and caffeine metabolism to determine whether tolerance to these substances contributes to anxiety. The collected data were used in a genome-wide association study and a polygenic risk score model of increased anxiety levels (Figure 1).

## 2 Methods

### 2.1 Study design and participants

In this study, we used data from an epidemiological study (conducted in 2020–2021) of 30,063 randomly selected adults at least 18 years of age registered in outpatient medical facilities in 8 federal districts of Russia. The regional distribution of participants is shown in Supplementary Table S1.

Whole blood samples were collected from all participants for genome-wide sequencing. The Hospital Anxiety and Depression Scale (HADS) and its HADS-A subscale (anxiety) were used to measure psycho-emotional well-being and anxiety levels. Based on the participants’ responses, medical professionals filled out the questionnaires. The original instructions by Zigmond et al. indicate that a HADS-A score of 8–10 is a borderline case (sub-clinically/clinically insignificant); a HADS-A score of ≥11 is an abnormal case (clinical/clinically significant). However, a series of HADS sensitivity studies has shown that the interpretation of HADS-A scores may vary depending on the disorder (11, 12). Therefore, to avoid overdiagnosis, only participants with a score of ≥11 were classified as showing clinically significant anxiety symptoms. Despite the subclinical anxiety symptoms, those who scored ≤10 were not considered to have anxiety. In some cases, the total score from the HADS was used to measure psychological distress and overall stress.

To analyze alcohol consumption, the participants were divided into three groups based on their drinking behavior and signs of alcohol abuse: Group 1 abstained from alcohol; Group 2 drank alcohol but showed no signs of alcohol abuse; and Group 3 drank alcohol (once a month or more) and displayed signs of alcohol abuse, such as uncontrolled intake, withdrawal syndrome, inability to meet personal and professional commitments, feeling shameful and guilty, alcohol-induced blackouts, etc.

Physical activity was assessed using the Global Physical Activity Questionnaire (GPAQ). The GPAQ measures the intensity, duration, and frequency in three domains of physical activity: work-related activity, travel to and from locations, and recreational activities. The participants’ weekly physical activity levels, or metabolic equivalent task units (METs), were calculated. In line with the WHO recommendations, participants with less than 600 METs a week were considered sedentary. The non-sedentary participants were divided into low-, moderate-, and high-intensity groups using the techniques described in previous studies (13, 14).

### 2.2 Ethical considerations

All participants gave their informed consent after full disclosure of the scope, methodology, and expected outcomes of the study. The study was approved by the local ethics committee of the Centre for Strategic Planning and Management of Biomedical Health Risks of the Federal Medical Biological Agency (FMBA) (Protocol No. 02, May 28, 2020).

### 2.3 DNA extraction and genome-wide sequencing

DNA was extracted from whole blood samples using the QIAamp DNA Mini Kit (Qiagen, Germany). A whole-genome sequencing (WGS) library was prepared using the Nextera DNA Flex kit (Illumina, USA), following the manufacturer’s instructions. The samples were sequenced to 150 bp reads with a minimum of 30× mean depth of coverage. The sequencing data from NovaSeq 6000 were demultiplexed using the bcl2fastq2 Conversion Software v2.20 (Illumina) (15). The quality of the generated FASTQ.GZ files was checked using FastQC v0.11.9, a quality control tool for high throughput sequence data (16). Only samples meeting the quality filtration criteria, such as distribution uniformity of nucleotides and G/C composition, were examined. The total size of the Undetermined*.fastq.gz files matched the size settings.

The reads were aligned to the reference genome (GRCh38.d1.vd1) using the Illumina Dragen Bio-IT platform (Illumina, USA). The quality of the generated BAM files was checked using the DRAGEN module of FastQC v0.11.9 (16), SAMtools v1.13 (17), and Mosdepth v0.3.1 (18). Only the samples meeting the quality filtration criteria, such as duplicates and unmapped reads, were examined. Strelka2 (v2.9.10) with default settings was used for small variant calling (19). Only variants with “PASS” status were used in further analyses. Variants violating the Hardy–Weinberg equilibrium (*p* < 10^−6^), multiallelic variants, and variants with a minor allele frequency of less than 1% were removed. Supplementary Table S4 provides information on the whole-genome sequence quality metrics.

### 2.4 Statistical analysis

Python 3.6.9 was used for data analysis. Relative and absolute value frequencies were calculated for categorical variables. Median values, standard deviations, and interquartile ranges were used for numerical variables.

The significance of associations between the variables and anxiety levels was tested by modeling a binary outcome for HADS scores using logistic regression (Statsmodels package). Sex and age were used as covariates.

A value of *p* of <0.05 was used as the significance threshold. Odds ratios were interpreted as the likelihood of developing anxiety. To address multiple testing, the Bonferroni adjustment (correction) was applied to all test results.

### 2.5 Genome-wide association study

Logistic regression was used to test genome-wide associations as a binary variable:


$$\log\left(\frac{p}{1-p}\right)=\beta_{0}+{\beta_{c}}^{\;*}\;C+{\beta_{g}}^{\;*}\;G$$

where β_0_ is the constant; β_c_ is the vector of the covariate effect; C is the covariate vector;

βg is the vector of the genotype effect; and G is the genotype vector.

Linear regression was used to test genome-wide associations as a continuous variable:


$$Y=\beta_{0}+{\beta_{c}}^{\;*}\;C+{\beta_{g}}^{\;*}\;G$$

where β_0_ is the constant; β_c_ is the coefficient of the covariate effect; C is the vector of the covariates; β_g_ is the vector of the genotype effect vector; and G is the genotype vector.

To account for population structure, Scikit-learn, a free machine learning package for the Python programming language, was used to perform a principal component analysis (PCA) on 15,000 SNPs from the Human Core Exome SNP Array (Illumina) with a minimum frequency of 5%. Over 50 simulations verified the stability of the results with less than 5% variability. The first 10 principal components were used as covariates in the genome-wide association studies. For variant annotation, we used dbSNP build 155.

Each position passing the filtering procedures was optimized. The target variable (anxiety) was encoded as a binary variable (1 for patients with anxiety). Calculations were performed using the Python library (statsmodels v0.12.2) parallelized on the Spark Cluster. Age, sex, and the first 10 principal components were used as covariates. After quality control filtration, 7,710,227 variants were tested. The variants passing a Bonferroni threshold of *p* < 5,0 × 10^−8^ were considered significant. The results were visualized using the LocusZoom JavaScript library. All significant variant sites were further analyzed for the quality of sequencing at each position. Information on the quality distribution is provided in the Supplement (Supplementary Figure S5).

### 2.6 Polygenic risk scores

For proper testing, 10% of the data were excluded from PRS modeling to be used as a test set in assessing the accuracy of the final model.

For the low-and high-anxiety groups, a polygenic risk score model was built using ridge regression in the scikit-learn library (Python v3.8). The regression minimizes the squared difference between the observed and predicted values and penalizes it with the sum of the squared coefficients:


$$\sum_{i}{\left(y_{i}-{\hat{y}}_{i}\right)}^{2}+\lambda \sum_{i}\;\beta_{j}^{2}$$

where y is the observed value; ${\hat{y}}_{i}$ is the estimated value; β is the ridge regression coefficient, and λ is the regularization parameter.

SNPs for the regression model were pre-selected based on their GWAS scores calculated as follows:


$$\text{score}=\frac{\left|\beta_{SNP}\right|}{p\;\cdot \;\left(\left|\beta_{\text{age}}\right|+\left|\beta_{\text{gender}}\right|+\left|\text{intercept}\right|\right)},$$

where $\beta_{SNP}$ is the SNP coefficient; $\beta_{\text{age}}$ is the coefficient of the age variable; $\beta_{\text{gender}}$ is the coefficient of the sex variable; intercept is the intercept of the model; and p is the value of *p* of the SNP.

The threshold for GWAS scores was chosen at random. SNPs passing the threshold were included in the model along with the following covariates: age, sex, and the first 10 principal components. To train the model, the data were divided into a training set (80%) and a validation set (20%). ROC AUC was calculated based on the performance of the model on the validation set.

The training procedure was repeated with a new threshold and set of SNPs. After a number of repetitions, the model with the highest ROC AUC on the validation set was selected as the final model. This approach allows for the selection of the optimal number of SNPs and provides the highest accuracy (20).

The accuracy of the final model, or ROC AUC, was tested on the test set.

## 3 Results

### 3.1 The study cohort

The study included 30,063 Russian adults aged 18 years and older (mean = 50 years) from 8 federal districts of Russia, who had no medical history of anxiety or other psychiatric disorders. Men made up 37.4% of the cohort (*n* = 11,232); women, 62.6% (*n* = 18,831). Detailed information on data completeness (sociodemographic factors, lifestyle, questionnaires, etc.) is presented in Supplementary Table S2.

#### 3.1.1 Sociodemographic factors of anxiety

##### 3.1.1.1 Anxiety prevalence

The mean HADS-A score was 6 points [3.0, 9.0]. Overall, 14% of the cohort had anxiety (≥ 11 points, HADS-A). The highest mean score was recorded in the North Caucasian Federal District (6 points); the lowest, in the Far Eastern, Northwestern, Siberian, and Ural Federal Districts (3 points). Supplementary Table S1 provides information on the overall level of stress and psychological distress (HADS) and the mean level of anxiety (HADS-A). Supplementary Figure S1 shows the distribution of HADS-A scores.

**Figure 2:**
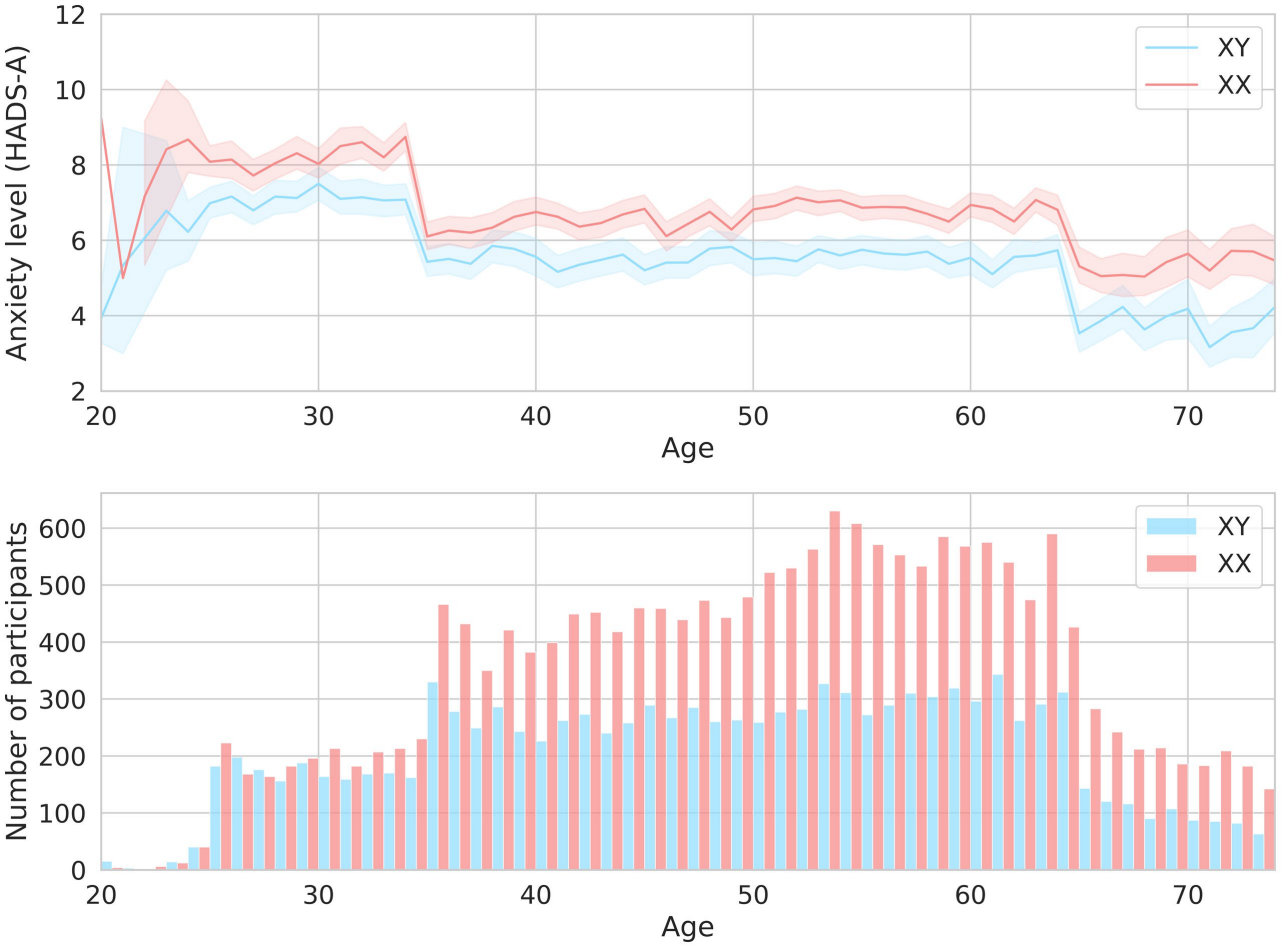
The mean HADS-A scores in men and women of different ages are shown in the upper graph. The men-to-women ratios in different age groups are shown in the lower graph.

Expectedly, sex and age were associated with anxiety levels. Clinical anxiety was twice as prevalent in women as in men (OR = 2.14, value of *p* = 9.06*10^−18^). Age had less of an effect (Table 1). Figure 2 illustrates how the mean HADS-A scores varied depending on age in both men and women. The 20–30-year age group had the highest mean score, which then gradually decreased.

**Table 1:** Sociodemographic factors significantly associated with increased anxiety levels (HADS-A).

	Low level (< 11 points), m [0.25; 0.75] or *n* (%) (*n* = 25,805)	High level (≥ 11 points), m [0.25; 0.75] or *n* (%) (*n* = 4,258)	OR	*р*-value
Age, years	50 [40; 59]	50 [39; 58]	0.92	8.8*10^−07^
Women, (%)	15,573 (60.3%)	3,258 (76.5%)	2.14	1.5*10^−87^
Men, (%)	10,232 (39.7%)	1,000 (23.5%)	0.47	1.5*10^−87^
Parental status*	No children (34%)	No children (16%)	1.02	1.65*10^−122^
	1 child (16.7%)	1 child (15.5%)	0.87	5.08*10^−03^
	2 children (46.5%)	2 children (66%)	2.22	4.33*10^−112^
	3 or more children (1.3%)	3 or more children (1.0%)	0.76	8.42*10^−02^
Marital status*	Never married (12.5%)	Never married (14.8%)	1.11	2.89*10^−02^
	Married (66.6%)	Married (60.2%)	0.86	1.44*10^−05^
	Divorced (12.8%)	Divorced (13.5%)	0.96	3.79*10^−01^
	Widower/widow (7.6%)	Widower/widow (10.8%)	1.40	1.11*10^−08^
Employment status*	Employed (59.6%)	Employed (58.9%)	0.90	5.68*10^−03^
	Never employed (10.1%)	Never employed (5.4%)	0.45	6.87*10^−26^
	Currently unemployed (5.4%)	Currently unemployed (10%)	1.89	1.31*10^−26^
	Retired (6.4%)	Retired (12.1%)	2.19	1.01*10^−43^
	Unemployed due to disability (1.0%)	Unemployed due to disability (2.6%)	2.9	1.22*10^−19^

*No adjustments were applied to age and sex significance. The significance of other parameters [marked with an asterisk (*)] was adjusted for age and sex.

Unsurprisingly, there was a strong association between marital status and anxiety levels. Anxiety levels were marginally lower in married participants. Widowhood increased anxiety levels by 40%, while divorce did not have such an effect. Although the number of children had a variety of effects, anxiety levels were markedly higher in the participants with two children (OR = 2.2, value of *p* = 4.33*10^−112^, Table 1).

Anxiety levels were also associated with employment: they were higher in the temporarily unemployed, unemployed due to disability, and non-working pensioners and almost three times as high in the unemployed due to a disability as in women.

Thus, there were significant associations between anxiety levels and marital, parental, and employment status. All sociodemographic factors significantly associated with anxiety are listed in Table 1.

#### 3.1.2 Associations between behavioral patterns and anxiety levels

##### 3.1.2.1 Sleep duration

**Figure 3:**
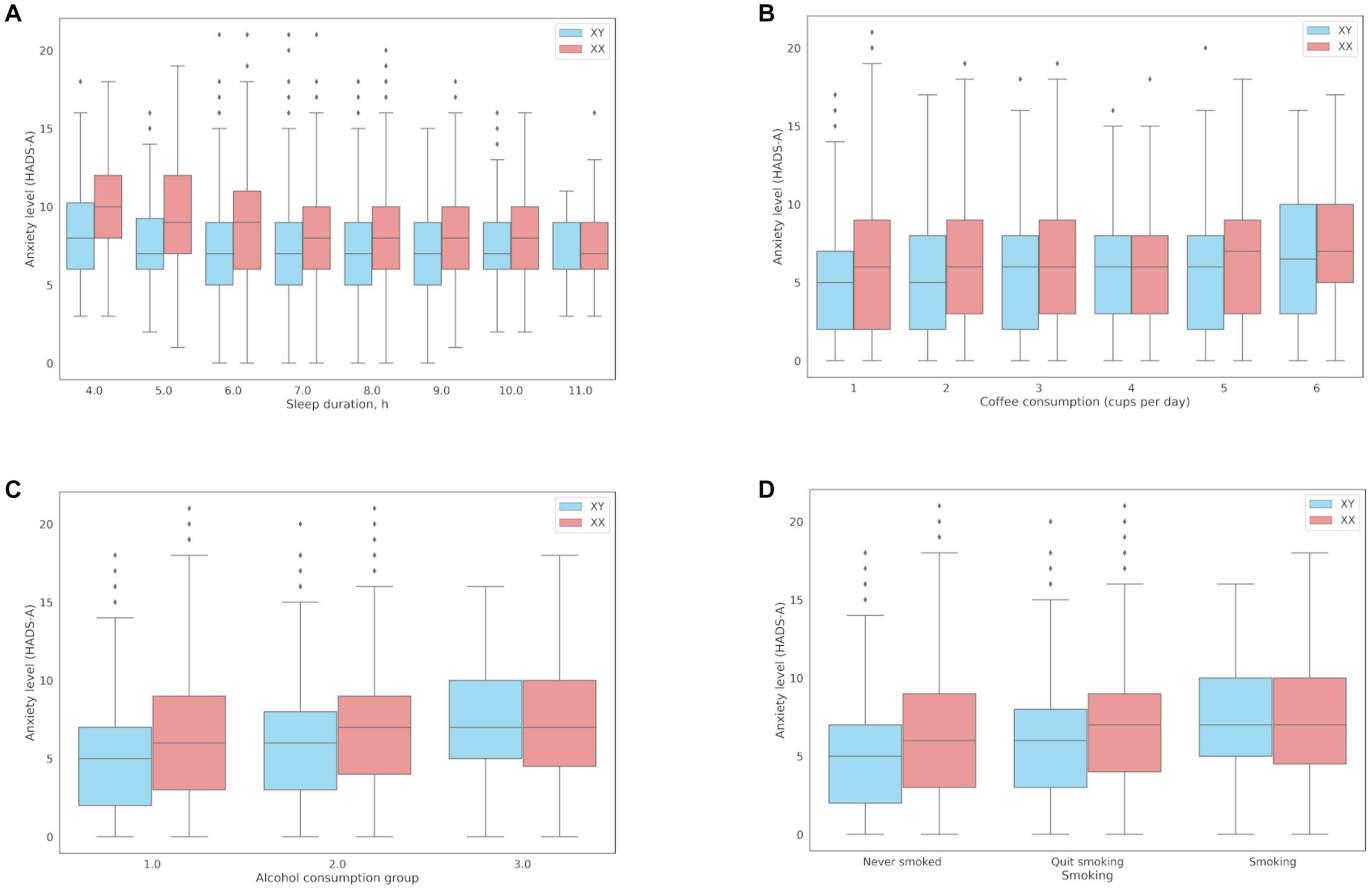
Associations between anxiety levels (HADS-A) and behavioral patterns: **(A)** Sleep duration; **(B)** Coffee consumption; **(C)** The median HADS-A anxiety scores in three groups of alcohol consumption: Group 1, median = 5 points [2.00, 9.00]; Group 2, median = 6 points [4.00, 9.00]; Group 3, median = 7 points [5.00, 10.00]; **(D)** Smoking.

The association between sleep and anxiety was examined in 16,518 participants who reported their average nightly sleep duration in the previous month. Expectedly, there was a negative association between sleep duration and anxiety (coef = −0.21; value of *p* = 1.2*10^−20^), even after adjustments for age and sex (coef = −0.2; value of *p* = 1.08*10^−29^). Sleep reduced by one hour resulted in a 13.5% increase in anxiety (OR = 0.865 [0.838, 0.891]). Figure 3A shows the associations between sleep duration and anxiety levels (HADS-A). Men experienced increased anxiety only when they slept less than 4 h, while women experienced subclinical anxiety (8–10 points, HADS-A) when they slept 6 h or less.

##### 3.1.2.2 Coffee consumption

The association between coffee consumption and anxiety was examined in 18,293 participants who reported their daily coffee consumption. Compared to the participants with lower anxiety levels, the participants with clinical anxiety (≥ 11 points) on average drank more cups of coffee per day (1.31 ± 1.4 and 1.23 ± 1.28, respectively; value of *p* = 2.70*10^−08^). There was a positive association between coffee consumption and anxiety levels (coef = 0.1254; OR = 1.13 per cup; value of *p* = 2.70*10^−08^). However, the effects of coffee consumption were somewhat reduced after adjustments for age and sex (coef = 0.0686, OR = 1.07, value of *p* = 2.33*10^−03^). Figure 3B shows the associations between daily coffee consumption and anxiety levels.

##### 3.1.2.3 Alcohol consumption

The association between alcohol consumption and anxiety was assessed in 26,864 participants who reported their alcohol consumption. Anxiety levels were more than twice as high in the participants who showed signs of alcohol abuse (ОR = 2.3; value of *p* = 6.3*10^−09^). The results are presented in Table 2.

**Table 2:** Mean HADS-A anxiety scores in participants with different alcohol consumption patterns.

	Group 1: no alcohol (*n* = 8,951)	Group 2: alcohol consumption, no signs of abuse (*n* = 17,578)	Group 3: alcohol consumption, with signs of abuse (*n* = 335)
Mean HADS score, ± standard deviation	10.34 ± 7.1	10.34 ± 6.01	13.08 ± 6.87
Mean HADS-А score, ± standard deviation	5.72 ± 4.13	6.34 ± 3.62	7.24 ± 3.59
р-value (of the difference between the mean score in the entire sample and in each group)	2.50*10^−02^	6.00*10^−15^	3.98*10^−07^

The mean HADS scores were identical in Groups 1 and 2 and matched the mean score in the entire cohort (10 points); the mean HADS score in Group 3 was higher (13 points). Meanwhile, the mean HADS-A scores varied across all three groups, with the lowest in Group 1 and the highest in Group 3, which was also higher than the mean score in the entire cohort. Anxiety levels in Groups 1–3 are shown in Figure 3C.

##### 3.1.2.4 Smoking

The association between smoking and anxiety levels was examined in all participants (Table 3). There was a positive correlation between smoking and anxiety (OR = 1.15). The lowest anxiety levels were found in lifelong non-smokers.

**Table 3:** Association between smoking and anxiety levels, adjusted for age and sex.

Low level (< 11 points), m [0.25; 0.75] or *n* (%) (*n* = 25,805)	High level (≥ 11 points), m [0.25; 0.75] or *n* (%) (*n* = 4,258)	OR	*p*-value
Lifelong non-smokers (64%)	Lifelong non-smokers (67%)	0.87 [0.80, 0.94]	3.65*10^−04^
Former smokers (16.6%)	Former smokers (14.4%)	1.05 [0.90, 1.23]	2.72*10^−01^
Current smokers (18.9%)	Current smokers (17.4%)	1.15 [1.05, 1.26]	3.16*10^−03^

Panel D in Figure 3 shows that anxiety levels were higher in smokers, including former smokers. This association, however, was more common in women.

##### 3.1.2.5 Physical activity

**Figure 4:**
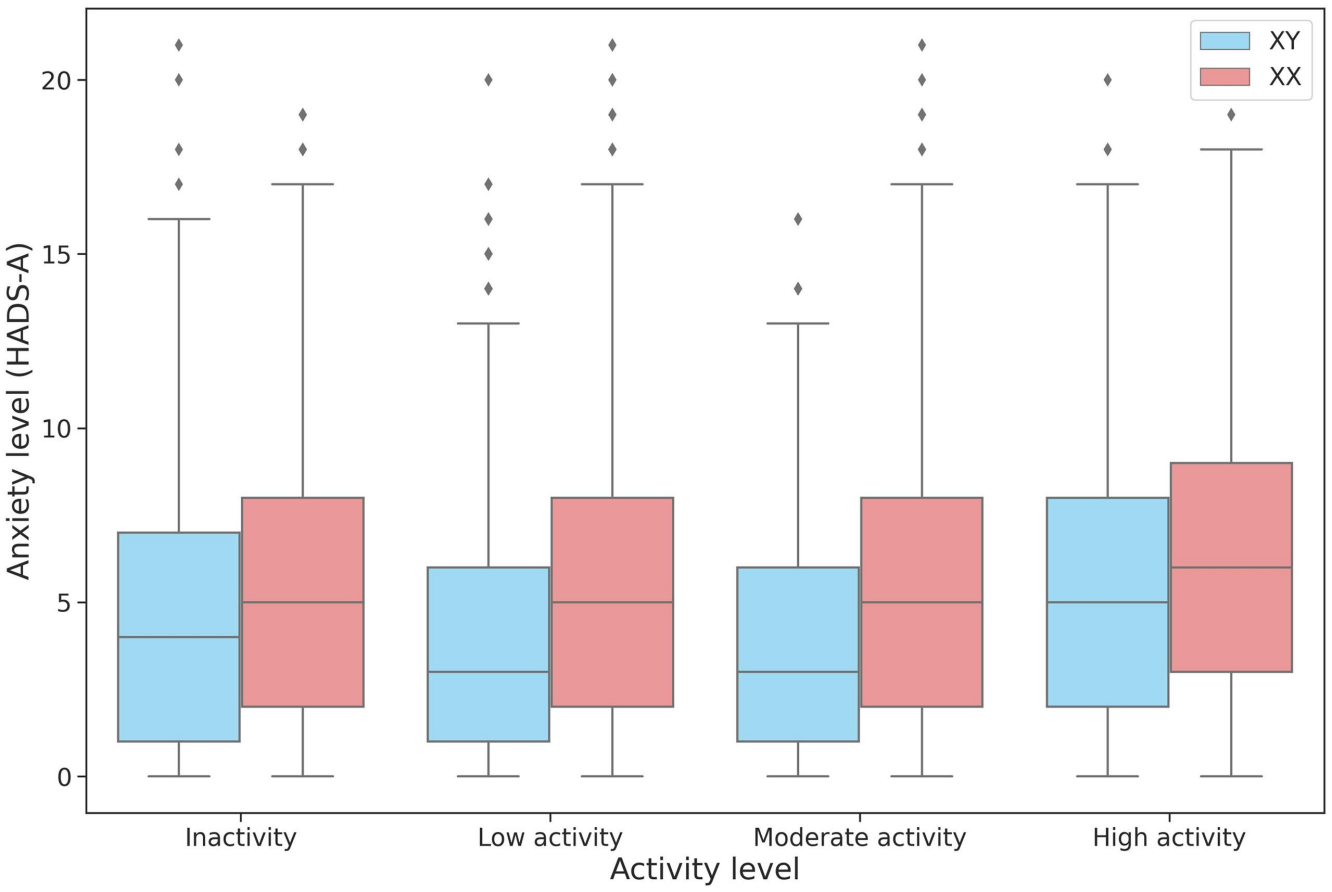
Changes in HADS-A scores depending on the intensity of physical activity in men and women.

The association between physical activity and anxiety levels was assessed in 27,320 participants. No linear relationship was found between the level of anxiety and physical activity. However, anxiety levels differed between the low-, moderate-, and high-intensity groups in both men and women (Table 4; Figure 4). The high-intensity group had the highest HADS-A scores.

**Table 4:** Association between physical activity and increased anxiety levels, adjusted for age and sex.

	Low level (< 11 points) (*n* = 24,651)	High level (≥ 11 points) (*n* = 2,669)	OR	Value of *p*
Sedentary (MET<600)	6,881 (27.9%)	826 (30.9%)	1.16	9.6*10^−4^
Low (MET 600–3,999)	10,296 (41.8%)	986 (36.9%)	0.78	1.16*10^−8^
Moderate (MET 4000–7,999)	4,159 (16.9%)	385 (14.4%)	0.84	0.0025
High (MET 8000+)	3,315 (13.4%)	472 (17.7%)	1.49	4.71*10^−13^

Additionally, we conducted a multivariate analysis of the following parameters: age, sex, number of children, marital status, alcohol consumption, coffee consumption, and sleep duration. The final model included all of the above parameters and generated a ROC AUC of 63.2% (Supplementary Figure S3). The multivariate and univariate analyses (Supplementary Figure S2) produced largely similar results, with minor differences due to a strong correlation between the parameters.

### 3.2 Specific genetic markers

Based on the behavioral patterns, we examined specific genetic markers of caffeine and alcohol metabolism and assessed their association with anxiety.

#### 3.2.1 Markers of caffeine metabolism

**Figure 5:**
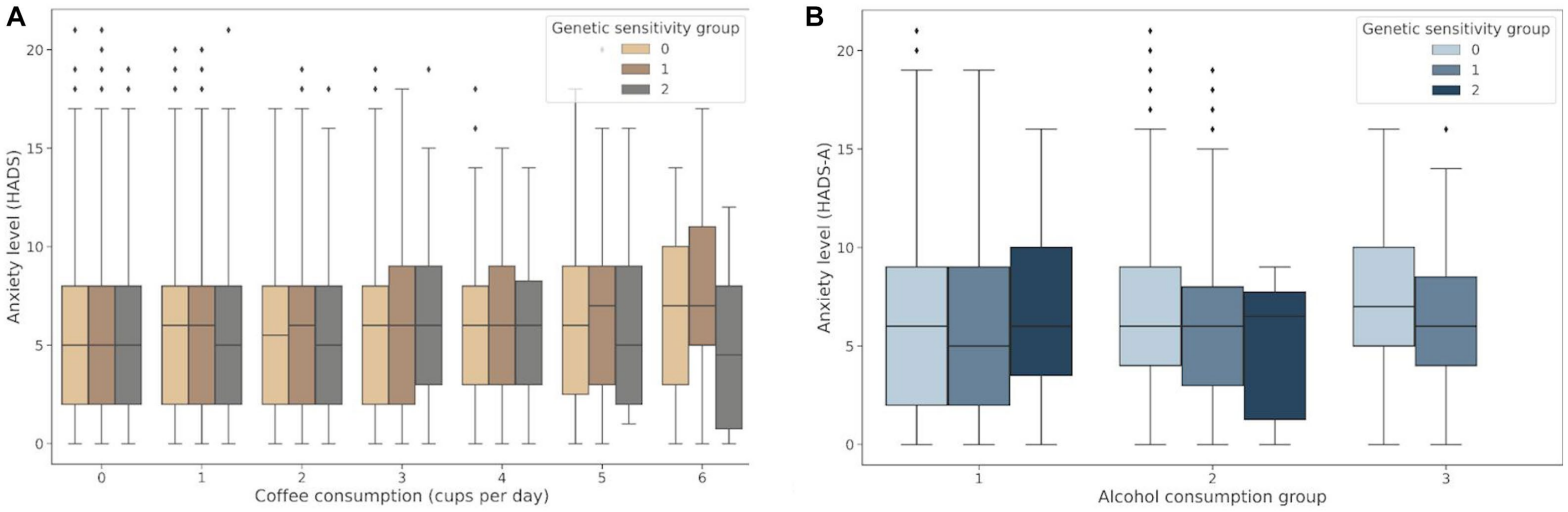
**(A)** Association between daily coffee consumption and anxiety levels in Groups 0–2; **(B)** Changes in anxiety levels in response to alcohol consumption in Groups 0–2: Group 0, no alcohol intolerance; Group 1, one marker of alcohol intolerance (rs671 or rs1229984); Group 2, two markers of alcohol intolerance (rs671 and rs1229984).

The genetic variants rs762551 (C > A) in *CYP1A2* and rs5751876 (T > C) in *ADORA2A* were examined as genetic markers of caffeine metabolism. According to the Pharmacogenomics Knowledge Base (PharmGKB), rs762551 АС and СС genotype carriers are “slower” caffeine metabolizers than АА genotype carriers, while rs5751876 TT genotype carriers are more likely to experience changes in anxiety levels in response to caffeine than СС and СТ genotype carriers. The participants were divided based on their caffeine intolerance: Group 0, no caffeine intolerance (rs762551 АА and rs5751876 СС or СТ); Group 1, carriers of one substitution (rs762551 АС or СС or rs5751876 ТТ); Group 2, carriers of two substitutions (rs762551 АС and СС and rs5751876 ТТ). The daily coffee consumption for each group is presented in Table 5. Figure 5A shows the effects of daily coffee consumption in these three groups. Anxiety levels were higher in the participants who consumed more coffee. However, this association was significant only in Group 1 (value of *p* = 4*10^−6^). It was insignificant in Group 2, probably due to the small number of participants. The significance of this association may increase for a larger sample.

**Table 5:** Daily coffee consumption in Groups 0–2.

Cups per day	Group 0 *n* = 9,874	Group 1 *n* = 6,770	Group 2 *n* = 1,649
0	3,356 (34%)	2,262 (33.4%)	561 (34%)
1	3,355 (34%)	2,247 (33.2%)	553 (33.5%)
2	1,744 (17.7%)	1,234 (18.2%)	289 (17.5%)
3	830 (8.4%)	585 (8.6%)	133 (8.1%)
4	300 (3%)	224 (3.3%)	60 (3.7%)
5	184 (1.8%)	119 (1.8%)	33 (2%)
6	105 (1.1%)	99 (1.5%)	20 (1.2%)

#### 3.2.2 Markers of alcohol metabolism

Both rs671 (G > A) in *ALDH2* and rs1229984 (T > C) in *ADH1B* were examined as markers of alcohol intolerance. The rs671 GG genotype has been linked to an increased risk of excessive alcohol consumption and alcohol dependence (21). However, this genotype is uncommon in the European population. Therefore, the rs1229984 CC genotype was also examined as another marker of predisposition to alcohol abuse, previously detected in the European population (22). The participants were divided based on their alcohol intolerance: Group 0, no alcohol intolerance (carriers of rs671 GG and rs1229984 CC); Group 1, carriers of one substitution associated with alcohol intolerance (rs671 AA or AG or rs1229984 TT or СТ); Group 2, carriers of two substitutions associated with alcohol intolerance (rs671 AA or AG and rs1229984 TT or СТ). Table 6 details alcohol consumption patterns in Groups 0–2. The graph in Figure 5B shows changes in the HADS-A scores in Groups 0–2 depending on the alcohol consumption patterns.

**Table 6:** Alcohol consumption patterns in Groups 0–2.

Alcohol consumption	Group 0 *n* = 22,792	Group 1 *n* = 4,047	Group 2 *n* = 25
Group 0, no alcohol	7,239 (31.8%)	1,697 (41.9%)	15 (60%)
Group 1, alcohol consumption, no signs of abuse	15,265 (67%)	2,303 (56.9%)	10 (40%)
Group 2, alcohol consumption, signs of abuse	288 (1.2%)	47 (1.2%)	0 (0%)

Alcohol consumption increased anxiety levels both in non-carriers and one substitution carriers (value of *p* = 1.48*10^−39^ and value of *p* = 4.8*10^−3^, respectively). However, anxiety levels were higher only in non-carriers with a tendency for alcohol abuse. Meanwhile, one substitution carriers had elevated anxiety levels even without signs of alcohol abuse. In two substitution carriers, anxiety levels also trended upward in response to alcohol consumption; however, this association was not significant, probably due to the small number of people in the group. There were no two substitution carriers showing signs of alcohol abuse.

The results of the analysis (Supplementary Table S3) suggest that caffeine/alcohol intolerance in combination with excessive alcohol/caffeine consumption may be linked to increased anxiety levels.

### 3.3 GWAS results

**Figure 6:**
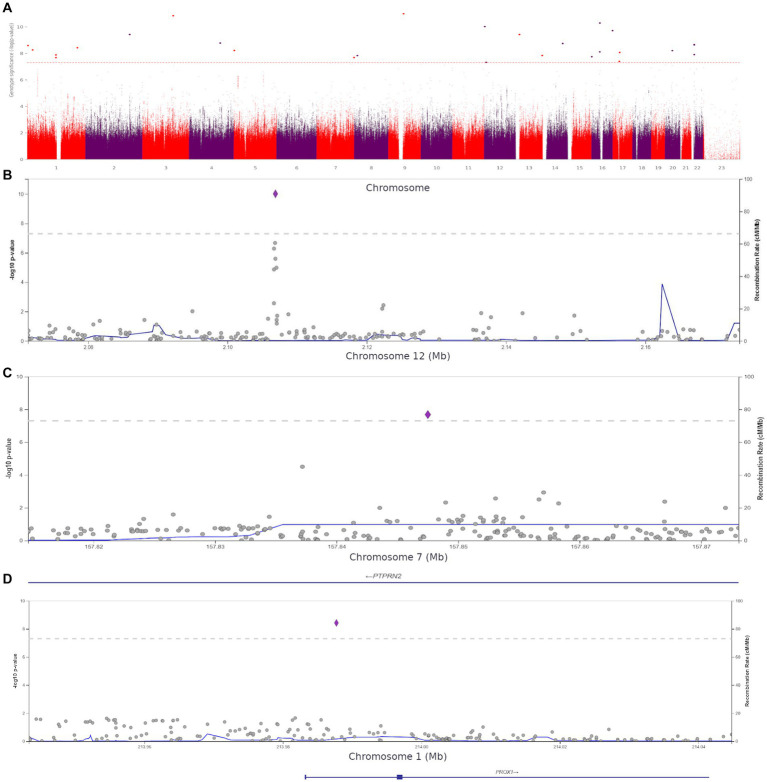
**(A)** Manhattan plot for associations between the detected variants and clinical anxiety (HADS-A, ≥11 points). The 5.0 × 10^−8^ and 1 × 10^−6^ significance thresholds are represented by gray dotted lines. **(B)** Association plot for the locus on chromosome 12 (chr12:2071357–2,173,517). The 5.0 × 10^−8^ significance threshold is represented by a gray dotted line. The SNP rs1205787230 with the strongest association with clinical anxiety is represented by a purple diamond. **(C)** Association plot for the locus on chromosome 7 (chr7:157814623–157,873,078). The 5.0 × 10^−8^ significance threshold is represented by a gray dotted line. The SNP rs3857647 with the strongest association with clinical anxiety is represented by a purple diamond. **(D)** Association plot for the locus on chromosome 1 (chr1:213943304–214,044,772). The 5.0 × 10^−8^ significance threshold is represented by a gray dotted line. The SNP rs941727476 with the strongest association with clinical anxiety is represented by a purple diamond. Linkage data were obtained from the 1,000 Genomes Project (1KGP). Color codes: red, r2 ≥ 0.8; yellow, r2 ≥ 0.6; green, r2 ≥ 0.4; blue, r2 ≥ 0.2; dark blue, r2 ≥ 0.0; gray, no data available.

We performed a genome-wide association study (GWAS) to assess the role of genetic variants in the development of anxiety. The comparison of the participants with clinical anxiety (≥ 11, HADS-A) and participants with no clinical anxiety (< 11, HADS-A) revealed 27 polymorphisms significantly associated with clinical anxiety (*p* < 5.0 × 10^−8^). These variants were located on different chromosomes (Figure 6A).

The Supplement (Supplementary Table S5) contains all variants with genome-wide significance. The discovered SNPs had not been previously associated with anxiety. Some of them were located in genes previously associated with several other psychiatric disorders, while others were in intergenic regions. For instance, rs1205787230 was found in the calcium channel gene *CACNA1C*. Loci containing significant variants are shown in Figures 6B–D.

**Figure 7:**
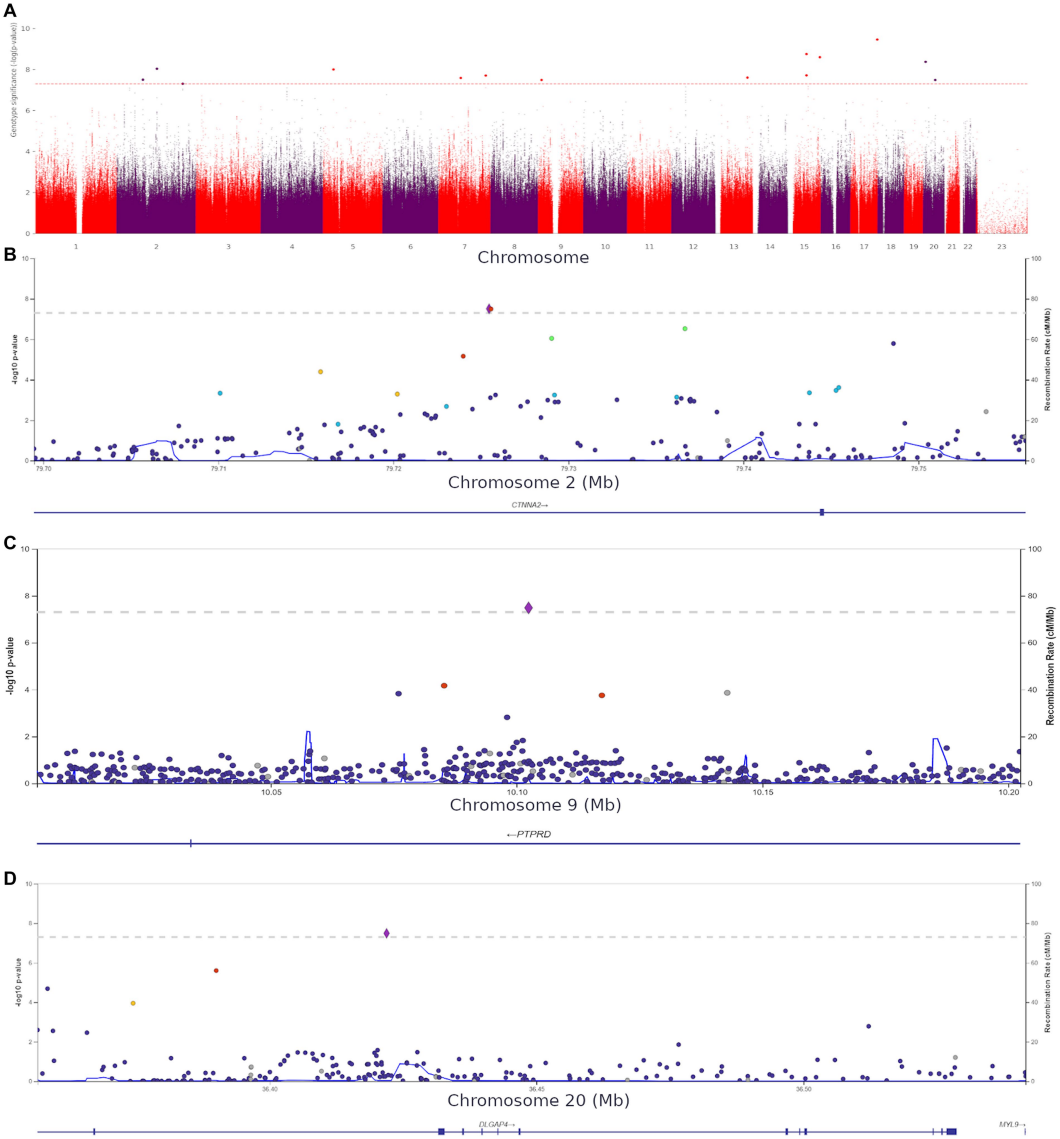
**(A)** Manhattan plot for associations between the detected variants and HADS-A scores. The 5.0 × 10^−8^ and 1 × 10^−6^ significance thresholds are represented by gray dotted lines. **(B)** Association plot for the locus on chromosome 2 (chr2:79699464–79,756,118). A 5.0 × 10^−8^ threshold for significance is represented by a gray dotted line. The SNP rs72927416 with the strongest association with clinical anxiety is represented by the purple diamond. **(C)** Association plot for the locus on chromosome 9 (chr9:10002424–10,202,424). The 5.0 × 10^−8^ significance threshold is represented by a gray dotted line. The SNP rs832264 with the strongest association with clinical anxiety is represented by a purple diamond. **(D)** Association plot for the locus on chromosome 20 (chr20:36356540–36,541,606). The 5.0 × 10^−8^ significance threshold is represented by a gray dotted line. The SNP rs832264 with the strongest association with clinical anxiety is represented by a purple diamond. Linkage data were obtained from the 1,000 Genomes Project (1KGP). Color codes: red, r2 ≥ 0.8; yellow, r2 ≥ 0.6; green, r2 ≥ 0.4; blue, r2 ≥ 0.2; dark blue, r2 ≥ 0.0; gray, no data available.

Using HADS-A scores as a continuous variable, we discovered 15 more variants (Supplementary Table S6) shown in Figure 7A.

We detected two *CTNNA2* polymorphisms significantly associated with high HADS-A scores: rs72927416 and rs7558324 (Figure 7B). Notably, many of the discovered variants, including one *DLGAP4* variant and *PTPRD* (rs832264) on chromosome 9, were associated with lower HADS-A scores, i.e., with the absence of clinical anxiety symptoms (Figures 7C,D).

These variants have not been previously linked to anxiety or psychiatric disorders. However, some of them might be crucial for better understanding the neurobiological basis of anxiety. The detection of *CACNA1C* rs1205787230 supports the hypothesis that calcium channels are involved in the development of increased anxiety in non-psychiatric patients. The detection of *PTPRN2*, which is required for the accumulation of noradrenaline, dopamine, and serotonin in the brain, adds to the evidence that monoamine neurotransmitters play a role in the development of anxiety. Moreover, some of the variants were located in genes directly or indirectly affecting neurogenesis and synaptic functions. Supplementary Figures S6, S7 show QQ plots and lambda values for both GWASs.

### 3.4 Polygenic risk score model of anxiety

**Figure 8:**
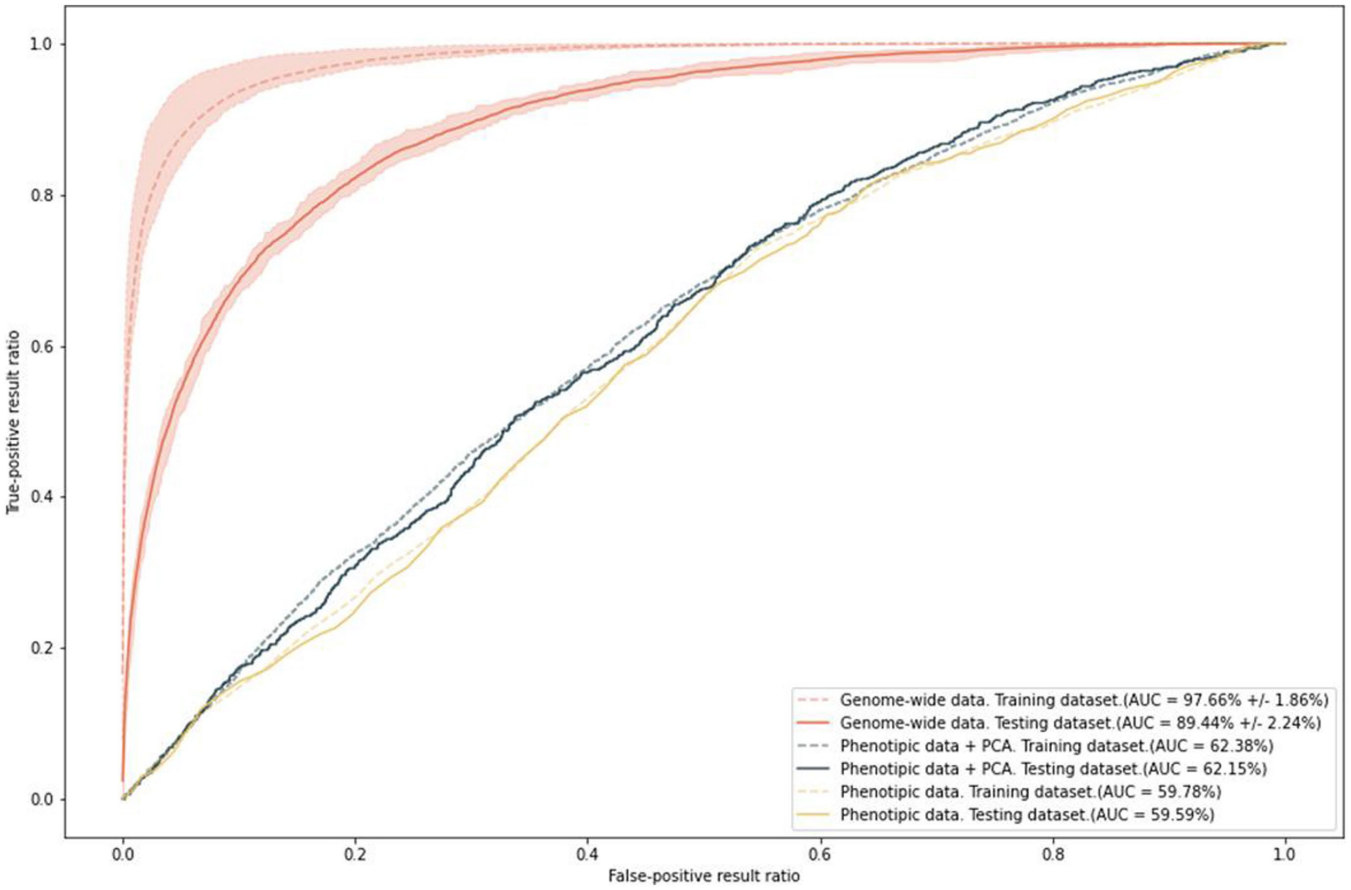
Results of PRS model training with anxiety as a binary variable; ROC AUC on the two types of data.

To predict the risk of increased anxiety (≥ 11 points, HADS-A), we built polygenic risk score (PRS) models. The ROC AUC of the final model was 97.66% ± 1.86% on the training set and 89.44% ± 2.24% on the test set (Figure 8). In addition, we built models based on two types of data: phenotypes, such as sex and age, which were used as covariates in the general model; and phenotypes combined with the first 10 principal components. This combination eliminated the effect of population structure. The accuracy values of these models were 59.15 and 61.38%, respectively.

The final model included 9,535 SNPs. Notably, the SNPs that did not reach the standard significant threshold in the GWAS had the largest “weights” in the model. These SNPs are listed in Supplementary Table S7.

The ROC AUC of the models on the test set slightly decreased. Nevertheless, the final model is extremely sensitive and specific for predicting the risk of anxiety. In contrast, age and sex, which are known to be significantly associated with anxiety, can only generate a prediction accuracy of about 60%.

## 4 Discussion

Anxiety is currently one of the most prevalent psycho-emotional disorders, associated with both genetic and non-genetic factors. This study assessed the prevalence of anxiety in Russia and examined its genetic architecture and risk factors.

We found associations between psycho-emotional well-being and sex, marital status, parental status, employment status, and lifestyle. According to the National Comorbidity Survey, anxiety is 1.5 times more common in women (23). In our study, anxiety levels were twice as high in women as in men. Studies on other populations have also found that social and marital well-being are associated with mental health (24). In married participants and participants with one child, anxiety levels were lower; in participants with two children and widows/widowers, anxiety levels were increased. Anxiety levels were higher among the unemployed, particularly due to disability or retirement.

Notably, young participants aged 20–30 had the highest level of anxiety. Previous epidemiological research has also shown that anxiety is more common in younger individuals (25). Participants in this age group were most likely going through a “critical period” of establishing themselves in their professional, personal, and social lives, which may have affected their psycho-emotional state.

Some unhealthy habits and lifestyle choices can affect psycho-emotional well-being. Alternatively, psycho-emotional distress can lead to lifestyle changes or addictions. Short sleep duration has been linked to reduced stress resilience and mood swings in healthy individuals (26). The National Institutes of Health (NIH) estimates that over 60% of sleep disorders in the United States are associated with increased levels of anxiety. In our study, there was an inverse association between sleep duration and anxiety levels. Women were more sensitive to a sleep deficit: ≤ 6 h of sleep per day increased anxiety to the subclinical level (HADS-A).

Smoking was another factor associated with increased anxiety, which is generally consistent with previous studies (27, 28). We found that increased anxiety was 15% more common in smokers and 13% less common in lifelong non-smokers. Importantly, anxiety levels were lower in former smokers.

We also found an association between anxiety and caffeine/alcohol consumption. Anxiety has been identified as a comorbid disorder or symptom of alcohol dependence in several studies (29). Caffeine has a very individual effect and can cause side effects, such as anxiety, even in safe amounts (30). In our study, alcohol consumption patterns indicative of alcohol abuse or dependence were associated with increased anxiety. However, the mean HADS-A score was also higher in alcohol consumers with no signs of abuse than in non-drinkers. Individuals prone to anxiety may turn to alcohol as a form of self-medication (31). Some authors believe that increased anxiety in alcohol abusers could be managed by treating their alcohol dependence (29). Notably, the participants with normal and increased anxiety levels did not show significant differences in the prevalence of genetic traits associated with alcohol intolerance caused by reduced enzymatic activity of human aldehyde dehydrogenase 2 (ALDH2). The study cohort did not include carriers of two substitutions with signs of alcohol abuse; nonetheless, the alcohol-consuming carriers of one substitution had increased anxiety even without signs of alcohol abuse. In non-carriers of intolerance-related variants, the mean HADS-A anxiety score increased only in response to alcohol abuse.

According to the Food and Drug Administration (FDA), 400 mg of caffeine per day, or 4–5 cups of coffee, is safe for most people. In our cohort, increased coffee consumption was associated with increased anxiety levels; however, the average daily coffee intake was still at an acceptable and safe level. It should be noted that several factors affect caffeine metabolism, processing, and excretion, including age, sex, hormones, and obesity. Genetic predisposition also significantly contributes to the rate of caffeine metabolism (32). Genetic variants associated with slow caffeine metabolism (33) have been linked to increased anxiety levels in response to coffee consumption (34). In our study, however, we found no compelling evidence that caffeine increased anxiety levels in carriers of the genetic markers of slow caffeine metabolism. Nonetheless, drinking four or more cups of coffee per day only marginally increased anxiety levels in all participants.

Many studies have examined the link between physical activity and mental health. However, the data on this subject is still very limited. Nevertheless, there is evidence that a sedentary lifestyle may be associated with anxiety (35). In our cohort, a sedentary lifestyle led to a 16% increase in anxiety levels. HADS-A scores showed an upward trend only in the male participants who engaged in high-intensity physical activity. Thus, the impact of daily physical activity and exercise is not straightforward and needs further research.

### 4.1 Genome-wide association study

We identified associations between genetic traits and anxiety in both binary and continuous trait tests. Although these genetic traits had not been associated with anxiety, some of them were within genes associated with other mental disorders and anxiety-like behavior in animal models.

Certain substitutions were of special interest since they were located within regions previously associated with mental disorders. We detected an SNP within a gene encoding subunits of calcium channels—*CACNA1C* (rs1205787230). The Cav1.2 channel, the α1C subunit of which is encoded by *CACNA1C*, has also been implicated in fear-related memory formation and pathway activation (36); however, its role in the brain and other tissues needs further research. *CACNA1C* SNPs have been repeatedly detected in studies on bipolar affective disorder (BAD) (37). However, functional magnetic resonance imaging (fMRI) revealed amygdala activation during emotional processing both in healthy carriers of the *CACNA1C* rs1006737 risk allele and people with BAD and schizophrenia (38). This finding is especially important, since amygdala subregions are known to play a critical role in mediating anxiety (39). When anxiety patients were exposed to a trigger, their amygdalae became hyper-activated (40). A recent study showed that impaired expression of *CACNA1C* disrupts spontaneous Ca2+ activity, leading to abnormal brain development and increased anxiety (41).

Another Ca2+ activity-associated gene, *PIEZO1* (rs371838333), also contained a significant SNP. The expression of PIEZO1 was studied in adult rats: in the cerebral cortex after ischemia/reperfusion injury (IRI) and in PC12 neuron-like cells after oxygen–glucose deprivation/reoxygenation (OGD/R) injury (42). The study found that the inhibition of *PIEZO1* could decrease intracellular Ca2+ concentrations following OGD/R. Hence, there is evidence that *PIEZO1* may be involved in brain injury via the activation of Ca2+/calpain signaling, while its abnormal expression may play a role in neuronal apoptosis after OGD/R.

Anxiety has traditionally been associated with the dysfunction of monoaminergic systems. Dopaminergic (DA), noradrenergic (NA), and serotonergic (5-HT) circuitries play an essential role in the neurobiology of major depression disorder (MDD) and anxiety disorders (43). We found no significant SNPs in the genes regulating monoamine neurotransmitters. However, there was a strong association between clinical anxiety and *PTPRN2* (rs3857647), which is required for noradrenaline, dopamine, and serotonin accumulation in the brain. Hypermethylated cytosine sites of *PTPRN2* have been linked to depression in trauma survivors (44).

We detected many significant genetic variants associated with HADS-A scores. For instance, STK24 (rs9517326), involved in excitatory synaptic transmission, was associated with higher scores. Previously, *STK24* overexpression was found to increase neuronal excitability in a hippocampal neuronal culture model. Moreover, *STK24* was identified as a possible binding partner of N-methyl-D-aspartate (NMDA) (glutamate receptor) in a study on epilepsy in mice (45).

High HADS-A scores in our study were significantly associated with two SNPs, *CTNNA2* (rs72927416), and *CTNNA2* (rs7558324). *CTNNA2* encodes a protein essential for normal cortical neuronal migration and neurite outgrowth. It is involved in intrauterine brain development and has been implicated in several psychiatric disorders, such as BAD (46), ADHD (47), alcoholism, schizophrenia, and general cognitive dysfunction (48). The detected variants have not been previously studied; other *CTNNA2* and *PTPRD* variants, however, have been linked to personality traits, such as impulsivity, schizophrenia, and autism, which manifest as unresponsiveness to visual stimuli (49). Alpha N-catenin is thought to be critical to synaptic contact stability and dendritic spine motility, which are crucial for neural plasticity. In mice, alpha N-catenin deficiency disrupts axon migration and causes abnormal startle responses and behaviors consistent with prepulse inhibition (50), which is believed to be an endophenotype of human psychosis. Monkeys classified as inhibited in infancy were more likely to be overly cautious in adulthood, while inhibited temperament (IT) was found to be hereditary. Moreover, an SNV with genome-wide significance was detected near *CTNNA2* (51).

Conversely, *PTPRD* (rs832264) was associated with lower HADS-A scores. This gene encodes the receptor-type protein tyrosine phosphatase, which interacts with several postsynaptic cell adhesion molecules via synapses. It has been associated with restless legs syndrome (RLS) (52) and psychoactive drug addiction. It has been hypothesized that lower *PTPRD* expression is associated with a reduced predisposition to psychoactive drug addiction, such as opioids (53). There is further evidence that *PTPRD* expression may affect the risk of Alzheimer’s disease and other neurodegenerative diseases (54, 55). Moreover, *PTPRD* variants have been recently implicated in schizophrenia (56, 57) and obsessive–compulsive disorder (OCD) (58). It is worth noting that *PTPRD* polymorphisms have been linked to mood instability based on the responses from the UK Biobank participants (59). In some cases, mood swings can be exacerbated by stress-related anxiety and persistent nervousness. Hence, the effects of the receptor-type protein tyrosine phosphatase on mental health are complex and should be studied further as a promising research topic.

*DLGAP4* (rs6039798) was another variant associated with lower HADS-A scores. *DLGAP4* encodes the membrane-associated guanylate kinase, which is localized in the postsynaptic density in neural cells. DLGAP proteins bind to three main glutamate receptors: NMDAR, AMPAR, and mGluR. They have been associated with numerous psychiatric and neurological disorders, including schizophrenia, OCD, and cerebellar ataxia (60). The role of glutamate in anxiety has been investigated in studies on the therapeutic effects of drugs that modulate its function (61). Pathological anxiety could also be caused by a GABA-glutamate imbalance leading to increased neuronal excitation (62). Supplementary Table S8 provides descriptions of other genes that contain significant associations. Functional changes in these genes might underlie many mental and neurological disorders and should be further studied. Their involvement in the development of anxiety also requires a more detailed examination.

### 4.2 Polygenic risk score

The genetic predisposition to anxiety is polygenic. The identified genes only partially explain the genetic risk. Therefore, we built a polygenic risk model of increased (clinical) anxiety that could be used to screen and identify risk groups. We analyzed 50 positions with the lowest *p*-values and selected the ones located in coding regions. The most significant were downstream gene variant *KLRB1* and some of the variants in the introns of *UBE2G1*, *SRRM4*, *TRIM2*, *SAMD12*, *SRRM4*, *SEMA5B*, *ANKFY1*, *WWOX*, *SLCO1B1*, *TENM3*, *MAGI2*, and *GSE1*.

*SRRM4* regulates neuronal differentiation and the splicing of protrudin gene transcripts, which are involved in neurite outgrowth during neurogenesis (63). Ubiquitin Conjugating Enzyme E2 G1 (UBE2G1) transcripts in the blood could be used as biomarkers of Alzheimer’s disease (64). The ubiquitin ligase TRIM2 is involved in axon growth, polarization, and specification and has neuroprotection properties. Changes in *TRIM2* function have been linked to progressive neurodegeneration in Alzheimer’s patients, while its variations have been associated with childhood-onset axonal neuropathy (65). Semaphorin 5B (*SEMA5B*) functions as a guidance cue and is essential for axon guidance and growth. Semaphorins have been linked to synaptic rearrangement (66). The SOX-6 transcription factor is involved in cortical interneuron differentiation. Cortical interneurons have been linked to several disorders, including epilepsy, autism, and schizophrenia; therefore, understanding the molecular basis of their variability may aid in developing new treatment options for these conditions (67). Variations in the scaffold protein coding gene MAGUK Inverted 2 (*MAGI-2*), which may be implicated in postsynaptic density and neurotransmission, have been found in patients with neurological disorders such as schizophrenia (68).

We also detected significant regions, which were cis-regulatory elements (CREs): chr1:244164789 (a promoter flanking region), which is located near *ZBTB18* and *ADSS*; *ZBTB18*, which encodes a DNA-binding transcription factor coordinating neurodevelopment during the formation of the mammalian cerebral cortex (69); *ADSS* (a catalyst in the early stages of adenosine monophosphate synthesis), the overexpression of which has been implicated in schizophrenia, suggesting that it plays an important role in the development of this condition.

## 5 Conclusion

The identification of the patterns linking lifestyle and genetic predisposition to psycho-emotional disorders and anxiety could provide new treatment and prevention options. Polygenic risk scores as part of screening procedures will enable the identification of high-risk individuals, which is crucial to developing individualized prevention strategies. In genetically predisposed individuals, lifestyle changes could be effective for both preventing and reducing anxiety.

## Data availability statement

The original contributions presented in the study are publicly available. This data can be found here: https://www.ebi.ac.uk/gwas/studies/GCP000797; accession number: GCP000797.

## Ethics statement

The studies involving human participants were reviewed and approved by the Local Ethics Committee of the Centre for Strategic Planning of FMBA of Russia (Protocol No. 02; May 28, 2020). The patients/participants provided their written informed consent to participate in this study.

## Author contributions

AY, DT, DK, AM, and VE: conceptualization ideas and validation. AR, OB, ES, MI, and SM: data curation. ES and MI: formal analysis and software. AA, AR, and DK: investigation. AY, DT, DK, VE, AM, and VY: methodology. VY, VM, AK, SK, and SY: project administration. AA, AR, and OB: resources. DK, VY, AK, SK, and SY: supervision. ES, MI, and MT: visualization. AY, DT, DK, AM, ES, VE, MI, LM, VY, and AR: writing – original draft and writing – review and editing. All authors contributed to the article and approved the submitted version.
