# Supplementary material for: Genetics of psycho-emotional well-being: genome-wide association study and polygenic risk score analysis
Source: Front Psychiatry. 2024 Jan 24;14:1188427. doi: 10.3389/fpsyt.2023.1188427 (PMC10847277; doi:10.3389/fpsyt.2023.1188427)

# Supplement

**Table S1.** Results of HADS survey in Federal Districts of Russia

|  | **Federal District (FD)** | **Mean HADS score (total) m [0.25; 0.75.** | **Mean HADS-А score (anxiety) m [0.25; 0,75.** | **Sample size** | **р-value (of the difference between the FD anxiety score and the mean anxiety score in the sample)** |
| --- | --- | --- | --- | --- | --- |
| 1 | Far Eastern Federal District | 5 [2, 8] | 3 [1, 6] | 4,023 | 1.45E^-30^ |
| 2 | Volga (Privolzhsky) Federal District | 4 [1, 7] | 4 [1, 7] | 2,426 | 5.12E^-97^ |
| 3 | Northwestern Federal District | 6 [3, 9] | 3 [1, 5] | 4,582 | 0.33 |
| 4 | Siberian Federal District | 7 [4, 10] | 6 [3, 9] | 3,712 | 1.57E^-10^ |
| 5 | Ural Federal District | 6 [4, 9] | 3 [1, 6] | 6,029 | 1.39E^-07^ |
| 6 | Central Federal District | 5 [3, 7] | 3 [0, 6] | 2,164 | 1.84E^-21^ |
| 7 | Southern Federal District | 6 [4, 9] | 4 [2, 7] | 4,757 | 0.0026 |
| 8 | North Caucasian Federal District | 8 [6, 10] | 5 [2, 8] | 2,370 | 7.51E^-141^ |

**Table S1.1. Regional** Distribution of participants

| 7 | Siberian Federal District | 1 (Altai Territory) | 1,487 |
| --- | --- | --- | --- |
| 3 | Southern Federal District | 3 (Krasnodar Territory) | 1,243 |
| 7 | Siberian Federal District | 4 (Krasnoyarsk Territory) | 1,483 |
| 8 | Far Eastern Federal District | 5 (Primorye Territory) | 1,869 |
| 4 | North Caucasian Federal District | 7 (Stavropol Territory) | 5 |
| 8 | Far Eastern Federal District | 8 (Khabarovsk Territory) | 19 |
| 2 | Northwestern Federal District | 11 (Arkhangelsk Region) | 1,495 |
| 1 | Central Federal District | 14 (Belgorod Region) | 360 |
| 3 | Southern Federal District | 18 (Volgograd Region) | 1,106 |
| 2 | Northwestern Federal District | 19 (Vologda Region) | 1,542 |
| 1 | Central Federal District | 20 (Voronezh Region) | 462 |
| 1 | Central Federal District | 24 (Ivanovo Region) | 1,653 |
| 4 | North Caucasian Federal District | 26 (Republic of Ingushetia) | 357 |
| 1 | Central Federal District | 28 (Tver Region) | 645 |
| 5 | Volga (Privolzhsky) Federal District | 36 (Samara Region) | 84 |
| 2 | Northwestern Federal District | 41 (Leningrad Region) | 515 |
| 7 | Siberian Federal District | 42 (Kemerovo Region) | 1,338 |
| 7 | Siberian Federal District | 50 (Novosibirsk Region) | 233 |
| 7 | Siberian Federal District | 52 (Omsk Region.) | 1,488 |
| 3 | Southern Federal District | 60 (Rostov Region) | 21 |
| 1 | Central Federal District | 61 (Ryazan Region.) | 1,102 |
| 8 | Far Eastern Federal District | 64 (Sakhalin Region) | 41 |
| 6 | Ural Federal District | 65 (Sverdlovsk Region) | 441 |
| 1 | Central Federal District | 70 (Tula Region) | 535 |
| 6 | Ural Federal District | 71 (Tyumen Region) | 1,417 |
| 6 | Ural Federal District | 74 (Yamal-Nenets Autonomous Area) | 254 |
| 6 | Ural Federal District | 75 (Chelyabinsk Region) | 52 |
| 8 | Far Eastern Federal District | 81 (Republic of Buryatia) | 1,309 |
| 4 | North Caucasian Federal District | 82 (Republic of Dagestan) | 1,019 |
| 4 | North Caucasian Federal District | 83 (Kabardino-Balkarian Republic) | 1,045 |
| 2 | Northwestern Federal District | 86 (Republic of Karelia) | 1030 |
| 5 | Volga (Privolzhsky) Federal District | 89 (Republic of Mordovia) | 711 |
| 4 | North Caucasian Federal District | 90 (Republic of North Ossetia-Alania) | 1286 |
| 5 | Volga (Privolzhsky) Federal District | 92 (Republic of Tatarstan) | 576 |
| 5 | Volga (Privolzhsky) Federal District | 97 (Chuvash Republic) | 1,055 |
| 8 | Far Eastern Federal District | 98 (Republic of Sakha (Yakutia)) | 785 |
|  |  | Total (all regions and territories) | 30,063 |

**Table S2.** Data completeness

| **Parameter** | **Filled values, count** | **Filled values, ratio** | **Missing values, count** | **Missing values, ratio** |
| --- | --- | --- | --- | --- |
| Age | 30,061 | 99.99% | 2 | 0.01% |
| Gender | 30,063 | 100.00% | 0 | 0.00% |
| HADS score | 30,063 | 100.00% | 0 | 0.00% |
| Anxiety status | 30,063 | 100.00% | 0 | 0.00% |
| Marital status | 29,964 | 99.67% | 99 | 0.33% |
| Children | 30,025 | 99.87% | 38 | 0.13% |
| Education | 30,015 | 99.84% | 48 | 0.16% |
| Proffession | 22,450 | 74.68% | 7,613 | 25.32% |
| Smoking status | 30,037 | 99.91% | 26 | 0.09% |
| Alcohol comsumption | 22,562 | 75.05% | 7,501 | 24.95% |
| Sleep duration | 16,518 | 54.94% | 13,545 | 45.06% |
| Physical activity | 29,991 | 99.76% | 72 | 0.24% |
| Coffee consumption | 18,367 | 61.10% | 11,696 | 38.90% |
| BMI | 29,973 | 99.70% | 90 | 0.30% |
| Alcohol abuse | 26,864 | 89.36% | 3,199 | 10.64% |

**Table S3.** Specific markers of coffee and alcohol metabolism

| Coffee consumption | rs762551 C> A | | | | |
| --- | --- | --- | --- | --- | --- |
|  | Homozygous reference allele (ref) | Heterozygous alleles | Homozygous alternative allele (alt) | Overall alt freuqncy | p-val (for alt) |
| High-anxiety participants (≥11 points, HADS-А) | 533 | 1,841 | 1,884 (44%) | 0.658642555 | 0.670070414 |
| Low-anxiety participants (<11 points, HADS-А) | 2,943 | 11,354 | 11,508 (44.6%) | 0.66595621 | 0.894195773 |
| Total sample | 3,476 | 13195 | 13,392 (44.5%) | 0.66492334 |  |
| Coffee consumption | rs5751876 T> С | | | | |
| High-anxiety participants (≥11 points, HADS-А) | 620 | 2,028 | 1,610 (37.8%) | 0.616251761 | 0.674142431 |
| Low-anxiety participants (<11 points, HADS-А) | 3,976 | 12,204 | 9,625 (37.2%) | 0.609455532 | 0.894878663 |
| Total sample | 4,596 | 14,232 | 11,235 (37.3%) | 0.6104181219 |  |
| Alcohol consumption | rs1229984 T> С | | | | |
| High-anxiety participants (≥11 points, HADS-А) | 31 | 538 | 3,689 | 0.929544387 | 0.69954488 |
| Low-anxiety participants (<11 points, HADS-А) | 251 | 3,582 | 21,972 | 0.920868049 | 0.903488735 |
| Total sample | 282 | 4,120 | 25,661 | 0.9220969298 |  |
| Alcohol consumption | Вариант в rs671 G> A | | | | |
| High-anxiety participants (≥11 points, HADS-А) | 4,242 | 16 | 0 | 0.001878816 | 1 |
| Low-anxiety participants (<11 points, HAD-А) | 25,707 | 97 | 1 | 0.001918233 | 1 |
| High-anxiety participants (≥11 points, HADS-А) | 29,949 | 113 | 1 | 0.0019126501 |  |
| Low-anxiety participants (<11 points, HADS-А) |  |  |  |  |  |

**Table S4**. Whole-genome sequence quality metrics for analyzed samples.

| **Parameter** | **Median [CI95]** |
| --- | --- |
| Total input reads | 960526432.00 [722512793.00, 1232555335.80] |
| Mapped reads | 959550303.00 [721480976.60, 1231768570.50] |
| Unmapped reads | 779015.00 [437996.60, 1365612.60] |
| Unmapped reads (%) | 0.08 [0.05, 0.14] |
| Estimated read length | 148.46 [146.61, 149.09] |
| Average sequenced coverage over genome | 45.81 [34.45, 58.69] |
| Median autosomal coverage over genome | 43.72 [33.03, 55.74] |
| Total variants | 4937926.00 [4883990.80, 4995616.70] |
| Aligned reads | 858652991.00 [650626674.10, 1094770633.50] |
| Average alignment coverage over genome | 43.03 [32.54, 54.82] |
| Coverage: mean (Qualimap) | 45.35 [34.02, 58.18] |
| Clipped reads, % (Qualimap) | 3.69 [3.01, 4.81] |
| Mean Mapping Quality (Qualimap) | 32.69 [31.90, 33.44] |
| Biallelic variants | 4566205.00 [4500426.50, 4635423.00] |
| Multiallelic variants | 373113.00 [312980.10, 422675.30] |
| Heterozygous | 3086464.00 [3007399.50, 3148178.80] |
| Homozygous | 1858300.00 [1793589.40, 1916357.60] |
| Het/Hom ratio | 1.67 [1.57, 1.74] |

**Table S5.** Results of testing genome-wide associations as binary variables. Polymorphisms associated with clinical anxiety (≥11 points, HADS-A) *

| **Chromosome** | **Position** | **Ref** | **Alt** | **Regression coefficient** | **р-value** | **The chi2-statistic** | **Hardy-Weinberg p-value** | **Gene code** | **SNP_id** | **Position within the gene** |
| --- | --- | --- | --- | --- | --- | --- | --- | --- | --- | --- |
| chr9 | 63764235 | T | G | 0.536991 | 1*10^-11^ | 9,186729 | 0.01012 | MYO5BP3 | rs77922510 | processed_pseudogene |
| chr3 | 130056468 | A | G | -0.5123 | 1.5*10^-11^ | 46,49148 | 8.03*10^-11^ | AC083906.4 | rs188291828 | unprocessed_pseudogene |
| chr16 | 34681324 | TA | T | 0.358715 | 5.2*10^-11^ | 52,08209 | 4.9*10^-12^ |  | rs1326929513 |  |
| chr12 | 2106887 | A | G | 0.380033 | 9.6*10^-11^ | 35,20311 | 2.27*10^-08^ | CACNA1C | rs1205787230 | protein_coding |
| chr16 | 88729697 | A | G | 0.364365 | 1.9*10^-10^ | 47,55998 | 4.7*10^-11^ | PIEZO1 | rs371838333 | protein_coding |
| chr2 | 187186370 | GC | G | 0.405354 | 3.8*10^-10^ | 25,76089 | 2.55*10^-06^ | AC007319.1 | rs1357371728 | lncRNA |
| chr13 | 16206744 | G | A | 0.247208 | 3.8*10^-10^ | 50,65913 | 9.99*10^-12^ |  | rs1170817771 |  |
| chr4 | 131717648 | CTT | C | 0.363386 | 1.7*10^-09^ | 37,11849 | 8.71*10^-09^ | RN7SL205P | rs139303903 | misc_RNA |
| chr14 | 85791755 | A | AT | 0.576448 | 1.8*10^-09^ | 3,910364 | 0.141539 |  | rs1434646593 |  |
| chr22 | 12370229 | C | A | 0.436973 | 2.2*10^-09^ | 14,72805 | 0.000634 |  | rs1385612084 |  |
| chr22 | 12370228 | C | G | 0.425705 | 2.3*10^-09^ | 16,7128 | 0.000235 |  | rs1453989645 |  |
| chr1 | 4078444 | G | A | 0.350092 | 2.6*10^-09^ | 7,588424 | 0.022501 |  | rs375437067 |  |
| chr1 | 213987718 | T | G | -0.78514 | 3.8*10^-09^ | 0,238232 | 0.887705 | PROX1 | rs941727476 | protein_coding |
| chr1 | 23837636 | A | G | 0.427433 | 5.5*10^-09^ | 14,42903 | 0.000736 | HMGCL | rs77857748 | processed_transcript |
| chr5 | 1291541 | T | G | 0.345116 | 6.1*10^-09^ | 40,9462 | 1.28*10^-09^ | TERT | rs55882184 | protein_coding |
| chr20 | 30423813 | TC | T | 0.349372 | 6.2*10^-09^ | 38,83346 | 3.69*10^-09^ |  | rs1229365098 |  |
| chr16 | 34253969 | GA | G | 0.327729 | 7.7*10^-09^ | 52,1909 | 4.64*10^-12^ |  | rs1170648539 |  |
| chr17 | 28737034 | TC | T | 0.461233 | 8.6*10^-09^ | 9,756617 | 0.00761 | NEK8 | rs1211099720 | protein_coding |
| chr22 | 12370232 | G | T | 0.435753 | 1.2*10^-08^ | 12,1935 | 0.00225 |  | rs1391156299 |  |
| chr1 | 122584036 | G | C | 0.261783 | 1.3*10^-08^ | 34,53635 | 3.17*10^-08^ |  | rs1162154200 |  |
| chr13 | 112750086 | T | TC | 0.256727 | 1.5*10^-08^ | 5,822853 | 0.054398 | ATP11A | rs530034081 | protein_coding |
| chr8 | 12662832 | C | T | 0.356334 | 1.5*10^-08^ | 31,21648 | 1.67*10^-07^ | AC068587.4 | rs186773460 | lncRNA |
| chr16 | 504633 | T | C | 0.292546 | 1.8*10^-08^ | 18,84502 | 8.09*10^-05^ | RAB11FIP3 | rs867764808 | protein_coding |
| chr7 | 157847512 | G | A | 0.266594 | 2.1*10^-08^ | 1,309495 | 0.519573 | PTPRN2 | rs3857647 | protein_coding |
| chr1 | 122584030 | T | A | 0.232391 | 2.1*10^-08^ | 11,10813 | 0.003872 |  | rs1443641020 |  |
| chr17 | 26936732 | TCGAAA | T | 0.319372 | 4.1*10^-08^ | 48,59805 | 2.8*10^-11^ |  | rs377401014 |  |
| chr12 | 7966052 | CAGG | C | 0.509854 | 4.8*10^-08^ | 4,57899 | 0.101318 | AC006511.7 | rs1195830461 | lncRNA |

** age and gender were used as covariates.*

**Table S6.** Results of the linear analysis of the genome-wide associations. Polymorphisms associated with HADS-A scores*

| **Chromosome** | **Position** | **Ref** | **Alt** | **Regression coefficient** | **р-value** | **The chi2-statistic** | **Hardy-Weinberg p-value** | **Gene code** | **SNP_id** | **Position within the gene** |
| --- | --- | --- | --- | --- | --- | --- | --- | --- | --- | --- |
| chr17 | 81207402 | G | A | -0.35844 | 3.46*10^-10^ | 7,319583 | 0.025738 | CEP131 | rs2456582 | protein_coding |
| chr15 | 57482422 | A | C | -0.38304 | 1.76*10^-09^ | 39,60905 | 2.51*10^-09^ | CGNL1 | rs2942035 | protein_coding |
| chr15 | 98221629 | A | G | -0.48413 | 2.51*10^-09^ | 7,690796 | 0.021378 | AC022523.1 | rs1442807 | lncRNA |
| chr20 | 7006252 | C | T | -0.47337 | 4.24*10^-09^ | 12,93536 | 0.001553 |  | rs6107908 |  |
| chr2 | 122511868 | A | G | -0.33492 | 9.19*10^-09^ | 1,701314 | 0.427134 | AC011246.1 | rs2049606 | lncRNA |
| chr5 | 30935228 | G | T | 0.230045 | 9.92*10^-09^ | 36,12406 | 1.43*10^-08^ |  | rs1276225 |  |
| chr15 | 57482142 | C | G | -0.3575 | 1.94*10^-08^ | 43,03305 | 4.52*10^-10^ | CGNL1 | rs1280384 | protein_coding |
| chr7 | 143981584 | T | C | 0.386548 | 1.99*10^-08^ | 46,95374 | 6.37*10^-11^ | OR2F1 | rs1034773 | processed_transcript |
| chr13 | 98499193 | T | G | 0.295919 | 2.5*10^-08^ | 38,93251 | 3.51*10^-09^ | STK24 | rs9517326 | protein_coding |
| chr7 | 67457587 | C | T | -0.46353 | 2.59*10^-08^ | 42,62362 | 5.55*10^-10^ |  | rs6460355 |  |
| chr2 | 79725467 | T | G | 0.303944 | 3.11*10^-08^ | 0,569704 | 0.752125 | CTNNA2 | rs72927416 | protein_coding |
| chr2 | 79725578 | T | G | 0.303656 | 3.19*10^-08^ | 0,56015 | 0.755727 | CTNNA2 | rs7558324 | protein_coding |
| chr20 | 36421920 | T | C | -0.37508 | 3.27*10^-08^ | 8,021675 | 0.018118 | DLGAP4 | rs6039798 | protein_coding |
| chr9 | 10102424 | C | T | -0.46731 | 3.27*10^-08^ | 27,31474 | 1.17*10^-06^ | PTPRD | rs832264 | protein_coding |
| chr2 | 201480753 | C | A | -0.34098 | 4.99*10^-08^ | 11,62145 | 0.002995 | STRADB | rs2110748 | protein_coding |

** age and gender were used as covariates.*

**Table S7.** Polygenic risk scores

| Chromosome | Position | Effect size | Effect significance | Gene | Gene variant | Coding/non-coding |
| --- | --- | --- | --- | --- | --- | --- |
| chr1 | 244164789 | -0,000188187 | 5,53583E-08 |  | regulatory_region_variant | promoter_flanking_region |
| chr1 | 157270665 | -0.000188187 | 5.53583E^-08^ | LINC02772 | upstream_gene_variant | lncRNA |
| chr19 | 42979717 | -0.000188187 | 5.53583E^-08^ | AC004784.1 | intron_variant&non_coding_transcript_variant | lncRNA |
| chr14 | 98237828 | -0.000188187 | 5.53583E^-08^ |  | intergenic_variant |  |
| chr12 | 9588782 | -0.167764077 | 3.47895E^-07^ | AC092821.4 | intron_variant&non_coding_transcript_variant | lncRNA |
| chr17 | 4300496 | -0.109784727 | 1.16671E^-06^ | UBE2G1 | intron_variant | protein_coding |
| chr12 | 9590931 | -0.158865199 | 1.24213E^-06^ | KLRB1 | downstream_gene_variant | protein_coding |
| chr5 | 84673190 | -0.251143821 | 1.63137E^-06^ |  | intergenic_variant |  |
| chr12 | 119040706 | -0.104308047 | 4.35541E^-06^ | SRRM4 | intron_variant | protein_coding |
| chr3 | 162209805 | -0.09370931 | 4.56882E^-06^ |  | intergenic_variant |  |
| chr3 | 162234703 | -0.09668946 | 4.64235E^-06^ |  | intergenic_variant |  |
| chr3 | 162224374 | -0.097028245 | 4.6874E^-06^ |  | regulatory_region_variant | open_chromatin_region |
| chr3 | 162235428 | -0.09645914 | 4.87678E^-06^ |  | intergenic_variant |  |
| chr3 | 162224875 | -0.09719711 | 5.13704E^-06^ |  | intergenic_variant |  |
| chr3 | 162223884 | -0.097759288 | 5.26334E^-06^ |  | intergenic_variant |  |
| chr3 | 162224690 | -0.101261014 | 5.70287E^-06^ |  | regulatory_region_variant | open_chromatin_region |
| chr8 | 101127520 | 0.196348356 | 6.03399E^-06^ | AP003469.1 | upstream_gene_variant | retained_intron |
| chr4 | 153221783 | 0.225427517 | 7.03896E^-06^ | TRIM2 | intron_variant | protein_coding |
| chr8 | 118292261 | -0.000131088 | 7.6519E^-06^ | SAMD12 | intron_variant | protein_coding |
| chr3 | 162230906 | -0.103835307 | 7.82733E^-06^ |  | intergenic_variant |  |
| chr3 | 193710705 | 0.334070106 | 8.05896E^-06^ |  | regulatory_region_variant | promoter_flanking_region |
| chr12 | 119041513 | -0.097018186 | 8.86481E^-06^ | SRRM4 | intron_variant | protein_coding |
| chr9 | 800773 | -0.229684685 | 9.84572E^-06^ |  | regulatory_region_variant | promoter_flanking_region |
| chr5 | 118423103 | 0.101523364 | 1.02007E^-05^ | LINC02208 | intron_variant&non_coding_transcript_variant | lncRNA |
| chr13 | 19073544 | 0.072221632 | 1.03149E^-05^ | AL137001.2 | downstream_gene_variant | lncRNA |
| chr5 | 118421048 | 0.101042393 | 1.05555E^-05^ | LINC02208 | intron_variant&non_coding_transcript_variant | lncRNA |
| chr12 | 119040055 | -0.099493849 | 1.07955E^-05^ | SRRM4 | intron_variant | protein_coding |
| chr13 | 76381502 | 0.237853263 | 1.09398E^-05^ |  | intergenic_variant |  |
| chr14 | 65243862 | -0.176978783 | 1.11244E^-05^ | AL355076.2 | intron_variant&non_coding_transcript_variant | lncRNA |
| chr3 | 123012066 | 0.082048911 | 1.11401E^-05^ | SEMA5B | intron_variant | protein_coding |
| chr3 | 123005314 | 0.089195119 | 1.11885E^-05^ | SEMA5B | intron_variant | protein_coding |
| chr9 | 107508060 | 0.158456981 | 1.2111E^-05^ |  | intergenic_variant |  |
| chr16 | 34827106 | 0.048575007 | 1.33088E^-05^ |  | intergenic_variant |  |
| chr3 | 122994284 | 0.085908729 | 1.33705E^-05^ | SEMA5B | intron_variant | protein_coding |
| chr11 | 16584554 | 0.277769056 | 1.38006E^-05^ | SOX6 | intron_variant&non_coding_transcript_variant | processed_transcript |
| chr17 | 4190376 | -0.107996663 | 1.53445E^-05^ | ANKFY1 | intron_variant | protein_coding |
| chrX | 115960495 | -0.185903807 | 1.58249E^-05^ | DANT2 | intron_variant&non_coding_transcript_variant | lncRNA |
| chr3 | 122994267 | 0.086396951 | 1.63777E^-05^ | SEMA5B | intron_variant | protein_coding |
| chr16 | 78186897 | 0.234653387 | 1.6463E^-05^ | WWOX | intron_variant | protein_coding |
| chr2 | 85488192 | -0.054748856 | 1.65279E^-05^ | RPSAP22 | downstream_gene_variant | processed_pseudogene |
| chr12 | 21197927 | 0.094516533 | 1.66499E^-05^ | SLCO1B1 | intron_variant | protein_coding |
| chr8 | 123745421 | 0.23441564 | 1.82244E^-05^ |  | intergenic_variant |  |
| chr5 | 118436597 | 0.105216472 | 1.82643E^-05^ | LINC02208 | intron_variant&non_coding_transcript_variant | lncRNA |
| chr4 | 182355633 | 0.248862099 | 1.84992E-05 | TENM3 | intron_variant | protein_coding |
| chr7 | 79343504 | 0.020045189 | 1.9935E-05 | MAGI2 | intron_variant | protein_coding |
| chr7 | 104247099 | 0.118804388 | 2.01541E^-05^ |  | intergenic_variant |  |
| chr5 | 118432157 | 0.119515085 | 2.08028E^-05^ | LINC02208 | intron_variant&non_coding_transcript_variant | lncRNA |
| chr18 | 10370599 | 0.198695735 | 2.14545E^-05^ | AP006219.1 | upstream_gene_variant | lncRNA |
| chr1 | 219447227 | 0.22607667 | 2.53171E^-05^ | LYPLAL1-AS1 | upstream_gene_variant | lncRNA |
| chr12 | 21197856 | 0.089241849 | 2.54884E^-05^ | SLCO1B1 | intron_variant | protein_coding |

**Table S8.** Functions of protein coding genes

| Index SNP | Protein coding genes | Potential association with neurological disorders |
| --- | --- | --- |
| rs2942035 | CGNL1 | Reliable synaptic communication in the CNS relies on strict homeostatic control around synapses [[65]](https://paperpile.com/c/kHV4n4/ZU6Q). This control is enabled by various regulatory mechanisms. including those operating at the blood–CSF barriers. Dysregulation and mislocalisation of tight junction membrane proteins. particularly claudins. increases the permeability of the blood–brain barrier (BBB) to proteins and immune cells. potentially leading to or exacerbating diseases. such as Alzheimer’s disease (AD). ischemic stroke. multiple sclerosis (MS) and schizophrenia [[66]](https://paperpile.com/c/kHV4n4/Vm2t).The CGNL1 gene encodes cingulin-like protein 1. also known as junction-associated-coiled-coil protein (JACOP). found in both adherens junctions and tight junctions. JACOP regulates the RhoA/ROCK signaling pathway required for the maintenance of cell-cell contacts. An activated RhoA/ROCK leads to claudin phosphorylation. which induces endothelial permeability. Studies have shown that 30% of individuals with deleted Claudin-5. or 22q11 deletion syndrome (22q11DS). suffer from schizophrenia. Levels of claudin-5 in the vessels of the hippocampus decreased in postmortem human brain tissue from schizophrenia patients; the mRNA and protein levels of claudins-5 and −12 and ZO-1 were associated with the age of schizophrenia onset and duration. Thus. it is safe to assume that an increased BBB permeability is associated with the development of the disease [66]. |
| rs941727476 | PROX1 | Prox1 *expresses* neural progenitor cells (NPCs) during the early stages *of* CNS development. Prox1 maintains the between the balance NPC proliferation and differentiation. Kaltezioti et al. hypothesize that Prox1 is a unique transcriptional regulator with a dual function: initially. it promotes cell cycle exit and NPC neuronal differentiation; then. suppresses neurite outgrowth by inhibiting Ca2+ signaling pathway [67]. The authors speculate that dual function provides enough time for nascent neurons to find the correct migratory route in the developing CNS environment before the initiation of mature neurite extension and axon elongation. |
| rs55882184 | TERT | The human telomerase reverse-transcriptase (TERT) gene is involved in telomere elongation. It has been detected in rodent neurons. Several studies have investigated its potential protective function in adult human brain. Spilsbury et al. studied TERT expression at different stages of Alzheimer's disease in situ and its protective property against oxidative damage in vitro [68]. The results showed that TERT-expressing hippocampal neurons did not contain the tau-protein associated with disease. Conversely. neurons expressing high levels of the associated tau protein. evidently. did not express TERT. These findings suggest that reduced TERT expression leads to an increased risk of oxidative damage in neurological conditions. |
| rs530034081 | ATP11A | ATP11A encodes integral membrane ATPase involved in phosphatidylserine *(*PtdSer*)* translocation. Segawa et al. investigated heterozygous point mutation in ATP11A in a patient affected by developmental delays and neurological deterioration; mice that carried the above mutation died of neurological disorders perinatally [69]. The mutation enables ATP11A to translocate PtdSer. which caused its leveled to drop in the outer leaflet of the plasma membranes and led to elevated sphingomyelin levels. Changes in phospholipid distribution led to cell growth. altered cholesterol homeostasis. and sensitivity to sphingomyelinase.A marked increase in SM levels was observed in the brains of mouse embryos with the ATP11A mutation. with subsequent neuronal defects and lethality. These findings demonstrate the physiological importance of the substrate specificity of plasma membrane flippases for the proper phospholipid distribution and. hence. proper cell growth and functioning. |
| rs867764808 | RAB11FIP3 | RAB11FIP3 is an GTPase effector protein involved in Rab/Arf-mediated endosomal recycling. GTPase effector proteins regulate different steps in vesicular traffic. such as docking. budding. fusion. and transport. Notably. endosomal trafficking in epithelial cells is not the only function of Rab11 interacting proteins (FIPs). Some studies report FIPs’ involvement in neuronal polarity [[70]](https://paperpile.com/c/kHV4n4/rdGZ) and migration during embryogenesis. Wang et al. [[71]](https://paperpile.com/c/kHV4n4/mZZb) showed FIPs’ involvement in maintain learning-related postsynaptic plasticity. Plasticity at excitatory synapses requires AMPA receptor mobilization and growth of dendritic spines. Moreover. in endosomes. Rab11-FIP2 serves as an adaptor for myosin Vb (MyoVb). Disruption or activation of this interaction leads to disruption or increased endosomal trafficking with the AMPAR pool to dendric spines. Hara et al. [[72]](https://paperpile.com/c/kHV4n4/Qfw8) demonstrated that disrupted endosomal trafficking caused impaired neuronal migration. These findings suggest that through the FIP3-dependent endosomal trafficking ADP ribosylation factor 6 (Arf6) regulates cortical neuronal migration in the intermediate zone. |

**Figure S1.** Distribution of HADS-A (anxiety) scores.

**
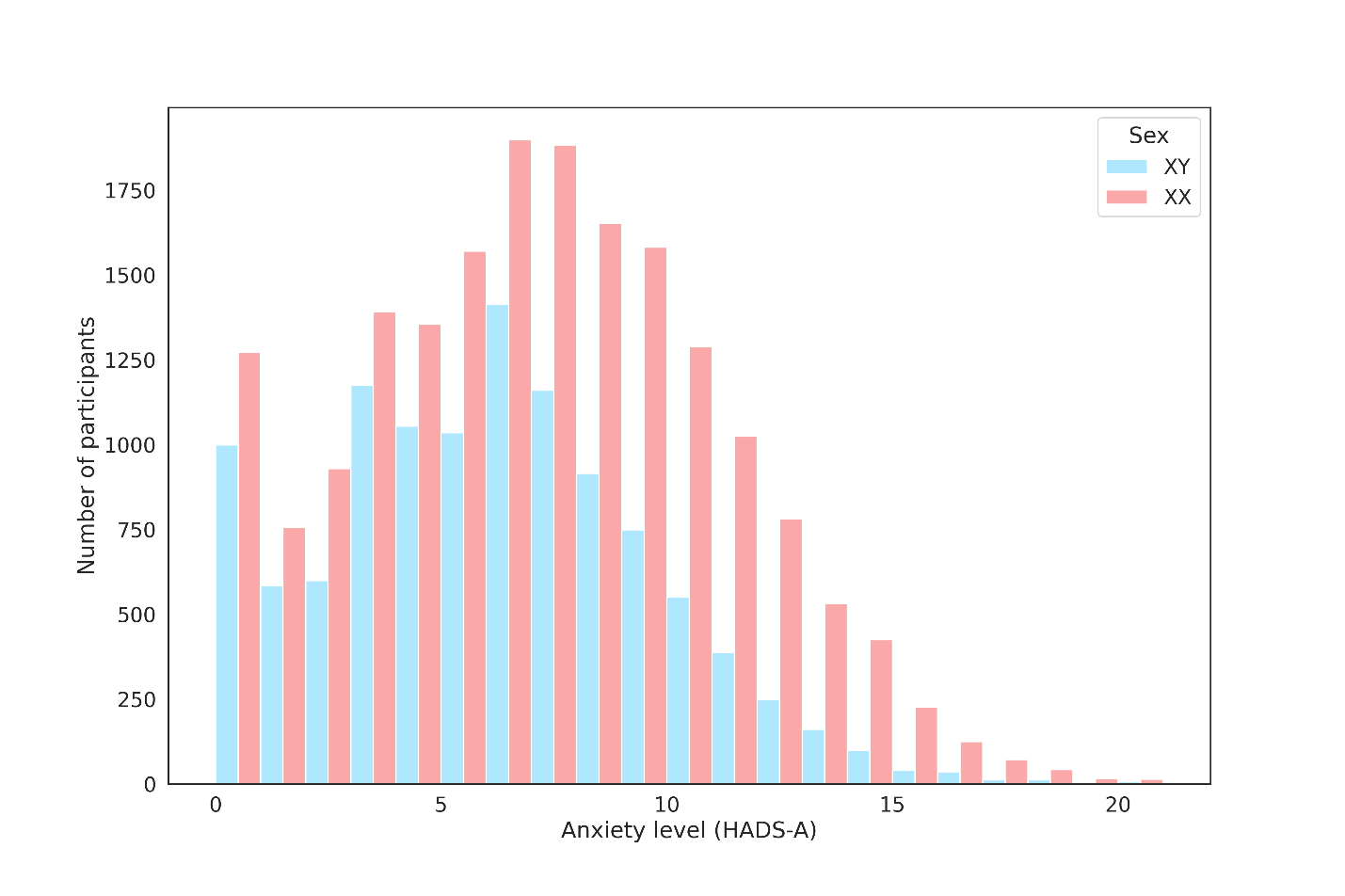
**

**Figure S2.** Multivariate analysis of parameters discussed in the study (sex was used as a covariate to make the model universally applicable).


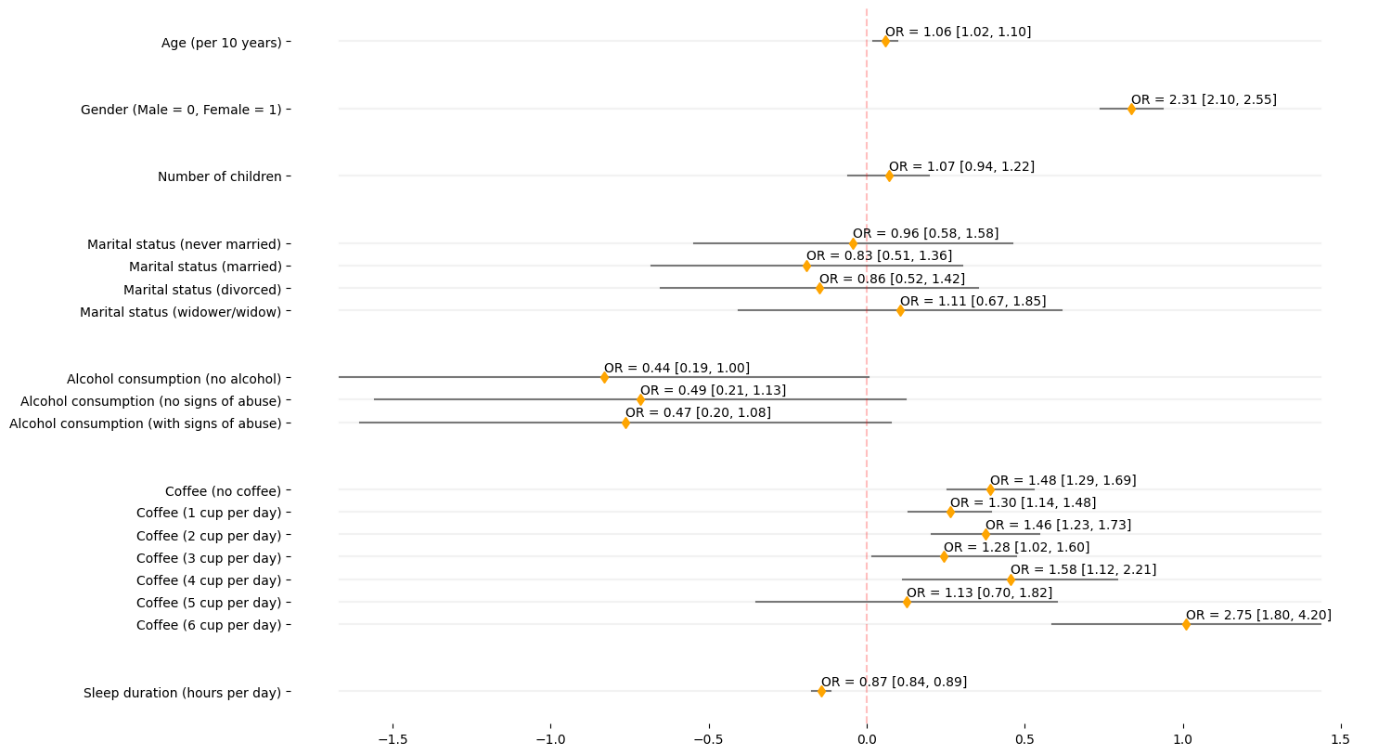


**Figure S3.** Multivariate model, ROC AUC = 63.2%

**
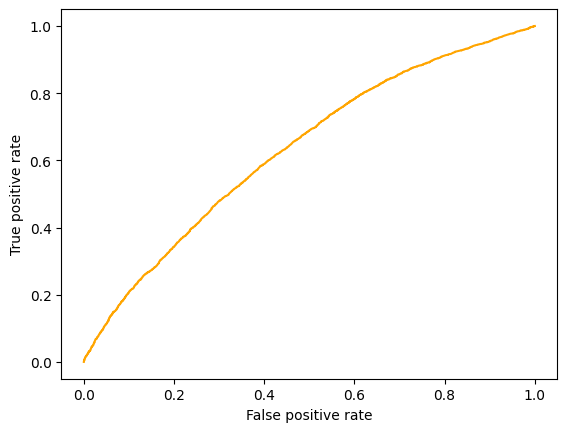
**

**Figure S4.** Forest plot for all associations found in the study

**
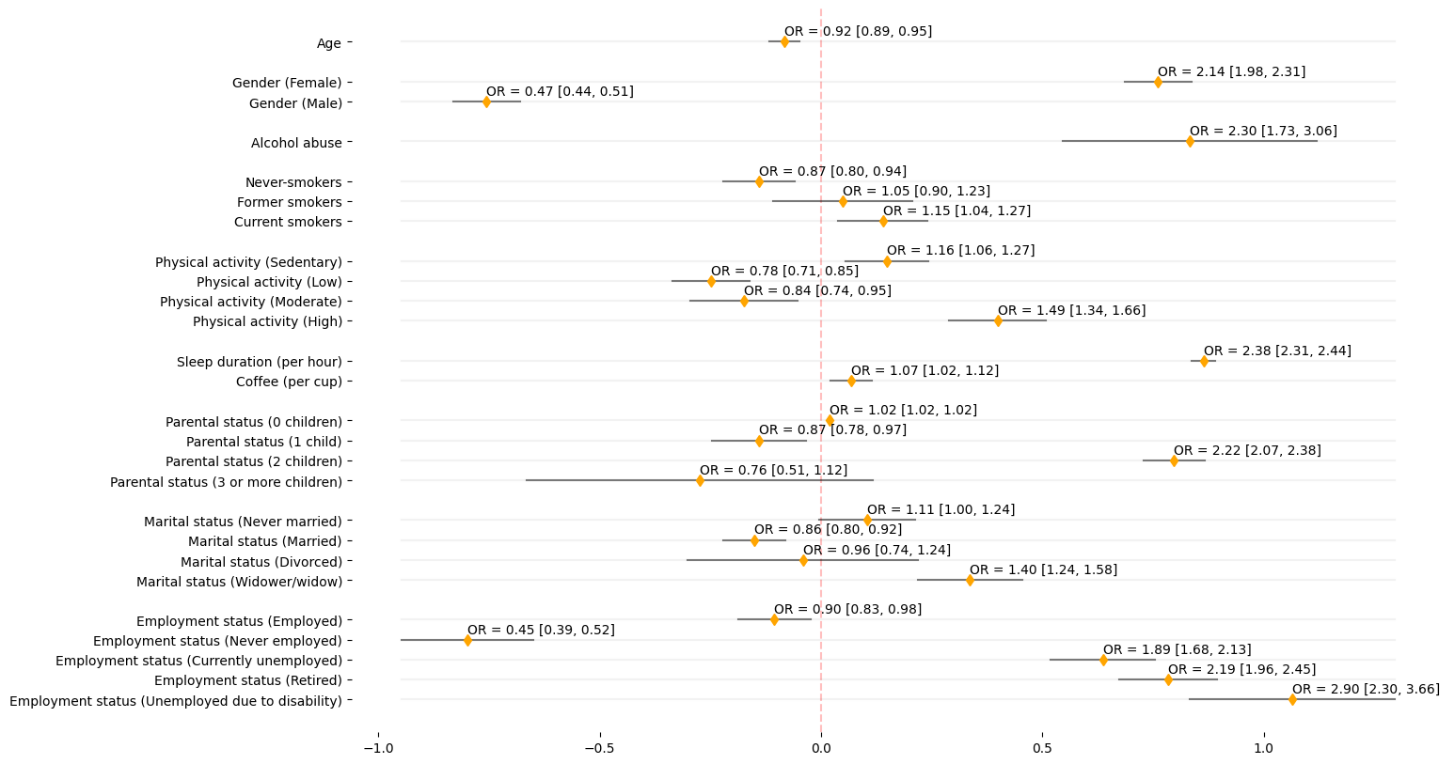
**

**Figure S5.** Quality distribution of variants

| **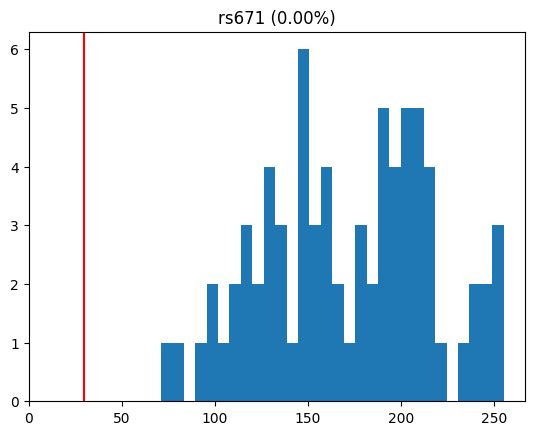** | **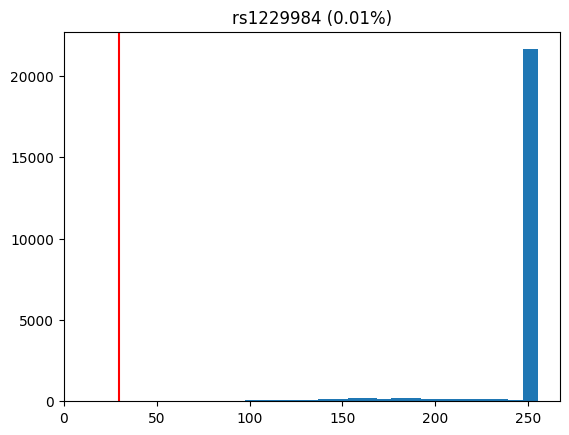** |
| --- | --- |
| **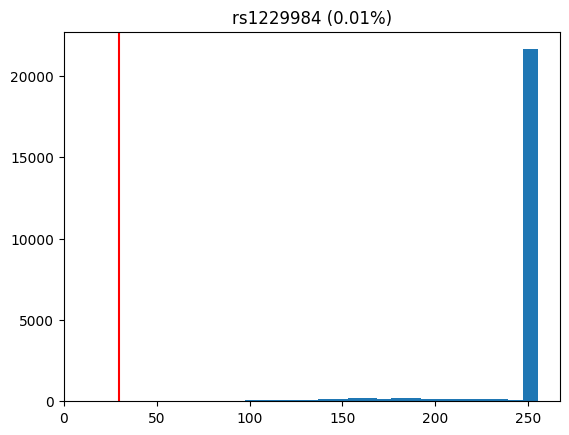** | **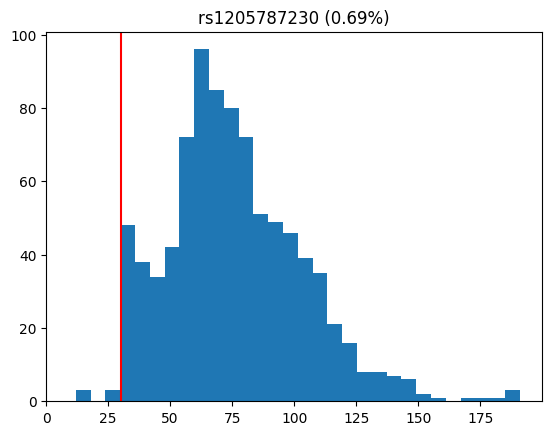** |
| **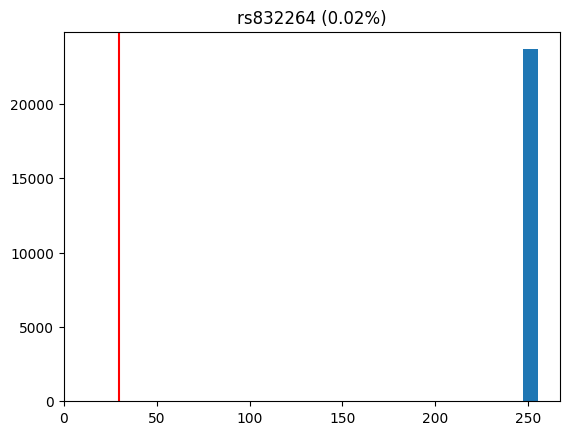** | **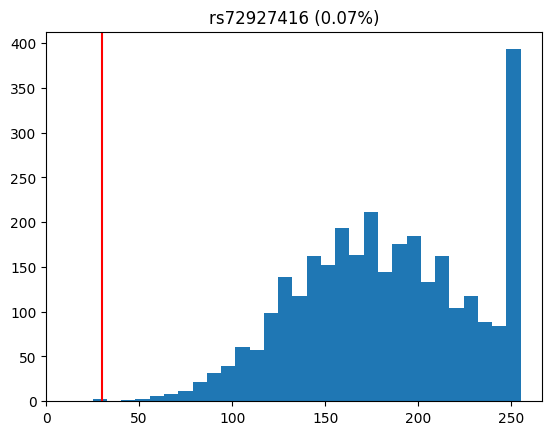** |
| **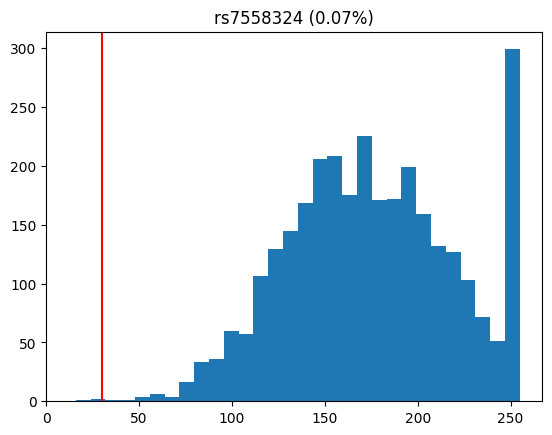** | **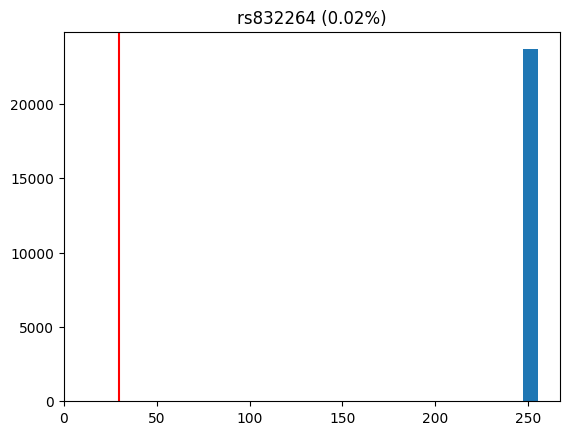** |

**Figure S6.**  QQ plots and lambda values for genome-wide associations tested as binary variables (clinical anxiety ≥11 points, HADS-A)


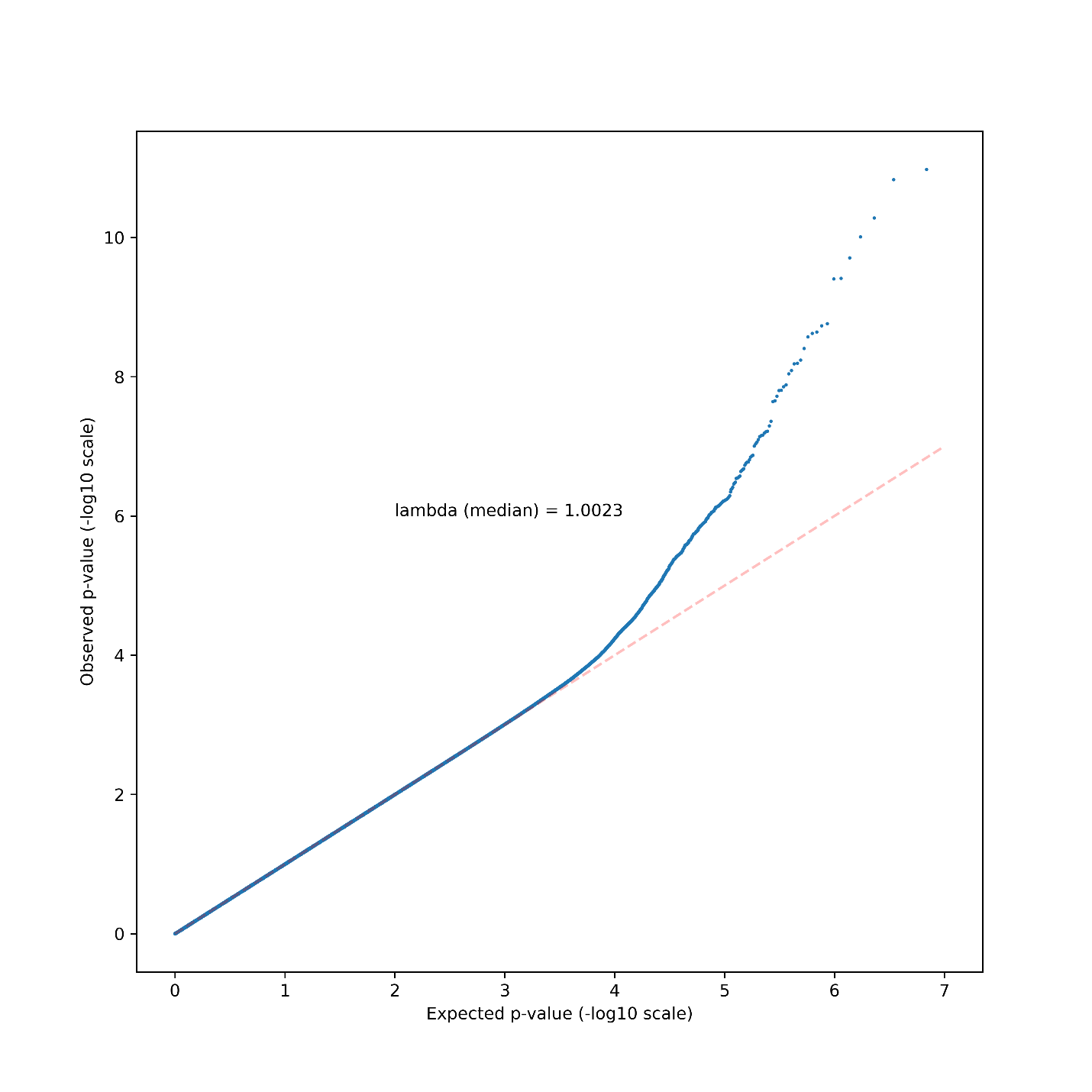


**Figure S7.**  QQ plots and lambda values for linear analysis of genome-wide associations


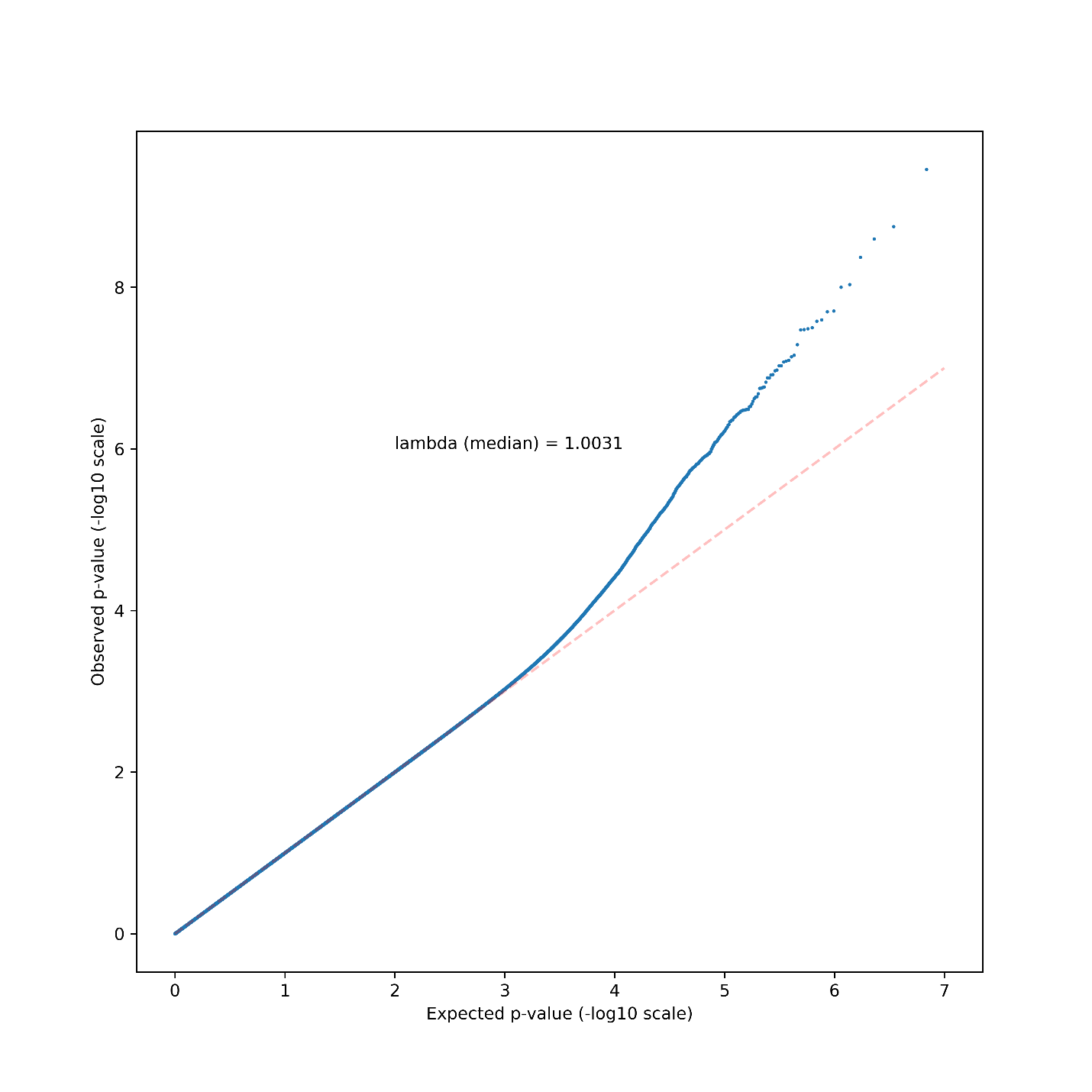

Supplement: Supplementary file 1 [file Data_Sheet_1.docx]
