# Supplementary material for: Genetics of psycho-emotional well-being: genome-wide association study and polygenic risk score analysis
Source: Front Psychiatry. 2024 Jan 24;14:1188427. doi: 10.3389/fpsyt.2023.1188427 (PMC10847277; doi:10.3389/fpsyt.2023.1188427)
Supplement: Supplementary file 2 [file Data_Sheet_2.docx]

**GWAS summary statistics**

Results of the genome-wide associations tested as a binary trait. Polymorphisms associated with clinical anxiety (≥11 points, HADS-A) *

| **Chromosome** | **Position** | **Ref** | **Alt** | **Regression coefficient** | **р-value** | **The chi2-statistic** | **Hardy-Weinberg p-value** | **Gene code** | **SNP_id** | **Position within the gene** |
| --- | --- | --- | --- | --- | --- | --- | --- | --- | --- | --- |
| chr9 | 63764235 | T | G | 0,536991 | 1*10^-11^ | 9,186729 | 0,01012 | MYO5BP3 | rs77922510 | processed_pseudogene |
| chr3 | 130056468 | A | G | -0,5123 | 1,5*10^-11^ | 46,49148 | 8,03*10^-11^ | AC083906.4 | rs188291828 | unprocessed_pseudogene |
| chr16 | 34681324 | TA | T | 0,358715 | 5,2*10^-11^ | 52,08209 | 4,9*10^-12^ |  | rs1326929513 |  |
| chr12 | 2106887 | A | G | 0,380033 | 9,6*10^-11^ | 35,20311 | 2,27*10^-08^ | CACNA1C | rs1205787230 | protein_coding |
| chr16 | 88729697 | A | G | 0,364365 | 1,9*10^-10^ | 47,55998 | 4,7*10^-11^ | PIEZO1 | rs371838333 | protein_coding |
| chr2 | 187186370 | GC | G | 0,405354 | 3,8*10^-10^ | 25,76089 | 2,55*10^-06^ | AC007319.1 | rs1357371728 | lncRNA |
| chr13 | 16206744 | G | A | 0,247208 | 3,8*10^-10^ | 50,65913 | 9,99*10^-12^ |  | rs1170817771 |  |
| chr4 | 131717648 | CTT | C | 0,363386 | 1,7*10^-09^ | 37,11849 | 8,71*10^-09^ | RN7SL205P | rs139303903 | misc_RNA |
| chr14 | 85791755 | A | AT | 0,576448 | 1,8*10^-09^ | 3,910364 | 0,141539 |  | rs1434646593 |  |
| chr22 | 12370229 | C | A | 0,436973 | 2,2*10^-09^ | 14,72805 | 0,000634 |  | rs1385612084 |  |
| chr22 | 12370228 | C | G | 0,425705 | 2,3*10^-09^ | 16,7128 | 0,000235 |  | rs1453989645 |  |
| chr1 | 4078444 | G | A | 0,350092 | 2,6*10^-09^ | 7,588424 | 0,022501 |  | rs375437067 |  |
| chr1 | 213987718 | T | G | -0,78514 | 3,8*10^-09^ | 0,238232 | 0,887705 | PROX1 | rs941727476 | protein_coding |
| chr1 | 23837636 | A | G | 0,427433 | 5,5*10^-09^ | 14,42903 | 0,000736 | HMGCL | rs77857748 | processed_transcript |
| chr5 | 1291541 | T | G | 0,345116 | 6,1*10^-09^ | 40,9462 | 1,28*10^-09^ | TERT | rs55882184 | protein_coding |
| chr20 | 30423813 | TC | T | 0,349372 | 6,2*10^-09^ | 38,83346 | 3,69*10^-09^ |  | rs1229365098 |  |
| chr16 | 34253969 | GA | G | 0,327729 | 7,7*10^-09^ | 52,1909 | 4,64*10^-12^ |  | rs1170648539 |  |
| chr17 | 28737034 | TC | T | 0,461233 | 8,6*10^-09^ | 9,756617 | 0,00761 | NEK8 | rs1211099720 | protein_coding |
| chr22 | 12370232 | G | T | 0,435753 | 1,2*10^-08^ | 12,1935 | 0,00225 |  | rs1391156299 |  |
| chr1 | 122584036 | G | C | 0,261783 | 1,3*10^-08^ | 34,53635 | 3,17*10^-08^ |  | rs1162154200 |  |
| chr13 | 112750086 | T | TC | 0,256727 | 1,5*10^-08^ | 5,822853 | 0,054398 | ATP11A | rs530034081 | protein_coding |
| chr8 | 12662832 | C | T | 0,356334 | 1,5*10^-08^ | 31,21648 | 1,67*10^-07^ | AC068587.4 | rs186773460 | lncRNA |
| chr16 | 504633 | T | C | 0,292546 | 1,8*10^-08^ | 18,84502 | 8,09*10^-05^ | RAB11FIP3 | rs867764808 | protein_coding |
| chr7 | 157847512 | G | A | 0,266594 | 2,1*10^-08^ | 1,309495 | 0,519573 | PTPRN2 | rs3857647 | protein_coding |
| chr1 | 122584030 | T | A | 0,232391 | 2,1*10^-08^ | 11,10813 | 0,003872 |  | rs1443641020 |  |
| chr17 | 26936732 | TCGAAA | T | 0,319372 | 4,1*10^-08^ | 48,59805 | 2,8*10^-11^ |  | rs377401014 |  |
| chr12 | 7966052 | CAGG | C | 0,509854 | 4,8*10^-08^ | 4,57899 | 0,101318 | AC006511.7 | rs1195830461 | lncRNA |

** age and gender were used as covariates.*

Results of the linear analysis of the genome-wide associations. Polymorphisms associated with HADS-A scores*

| **Chromosome** | **Position** | **Ref** | **Alt** | **Regression coefficient** | **р-value** | **The chi2-statistic** | **Hardy-Weinberg p-value** | **Gene code** | **SNP_id** | **Position within the gene** |
| --- | --- | --- | --- | --- | --- | --- | --- | --- | --- | --- |
| chr17 | 81207402 | G | A | -0,35844 | 3,46*10^-10^ | 7,319583 | 0,025738 | CEP131 | rs2456582 | protein_coding |
| chr15 | 57482422 | A | C | -0,38304 | 1,76*10^-09^ | 39,60905 | 2,51*10^-09^ | CGNL1 | rs2942035 | protein_coding |
| chr15 | 98221629 | A | G | -0,48413 | 2,51*10^-09^ | 7,690796 | 0,021378 | AC022523.1 | rs1442807 | lncRNA |
| chr20 | 7006252 | C | T | -0,47337 | 4,24*10^-09^ | 12,93536 | 0,001553 |  | rs6107908 |  |
| chr2 | 122511868 | A | G | -0,33492 | 9,19*10^-09^ | 1,701314 | 0,427134 | AC011246.1 | rs2049606 | lncRNA |
| chr5 | 30935228 | G | T | 0,230045 | 9,92*10^-09^ | 36,12406 | 1,43*10^-08^ |  | rs1276225 |  |
| chr15 | 57482142 | C | G | -0,3575 | 1,94*10^-08^ | 43,03305 | 4,52*10^-10^ | CGNL1 | rs1280384 | protein_coding |
| chr7 | 143981584 | T | C | 0,386548 | 1,99*10^-08^ | 46,95374 | 6,37*10^-11^ | OR2F1 | rs1034773 | processed_transcript |
| chr13 | 98499193 | T | G | 0,295919 | 2,5*10^-08^ | 38,93251 | 3,51*10^-09^ | STK24 | rs9517326 | protein_coding |
| chr7 | 67457587 | C | T | -0,46353 | 2,59*10^-08^ | 42,62362 | 5,55*10^-10^ |  | rs6460355 |  |
| chr2 | 79725467 | T | G | 0,303944 | 3,11*10^-08^ | 0,569704 | 0,752125 | CTNNA2 | rs72927416 | protein_coding |
| chr2 | 79725578 | T | G | 0,303656 | 3,19*10^-08^ | 0,56015 | 0,755727 | CTNNA2 | rs7558324 | protein_coding |
| chr20 | 36421920 | T | C | -0,37508 | 3,27*10^-08^ | 8,021675 | 0,018118 | DLGAP4 | rs6039798 | protein_coding |
| chr9 | 10102424 | C | T | -0,46731 | 3,27*10^-08^ | 27,31474 | 1,17*10^-06^ | PTPRD | rs832264 | protein_coding |
| chr2 | 201480753 | C | A | -0,34098 | 4,99*10^-08^ | 11,62145 | 0,002995 | STRADB | rs2110748 | protein_coding |

** age and gender were used as covariates.*
